# Behavioral Improvement and Task-Related Brain Activation Following Cognitive Training in Children/Adolescents and Young Adults: A Three-Level Behavioral and Coordinate-Based Neuroimaging Meta-Analysis

**DOI:** 10.3390/brainsci16080861

**Published:** 2026-08-14

**Authors:** Ting Fan, Chengzhen Liu, Yang Liu, Geng Li

**Affiliations:** 1 School of Elementary Education, Hunan First Normal University, Changsha 410205, China; 2 Faculty of Psychology, Southwest University, Chongqing 400715, China; 3 School of Sports Science, Jishou University, Jishou 416000, China; 4 School of Psychology, Research Center for Exercise and Brain Science, Shanghai University of Sport, Shanghai 200438, China

**Keywords:** cognitive training, behavioral improvement, task-based neuroimaging, meta-analysis, childhood, young adulthood

## Abstract

Background/Objectives: Cognitive training provides a framework for examining how repeated practice relates to changes in cognitive performance and task-related brain activation, yet age-stratified patterns of neural effects remain unclear. Methods: We conducted a three-level behavioral meta-analysis and a coordinate-based Seed-based Mapping (SDM) meta-analysis of task-based neuroimaging studies to jointly examine behavioral improvement and task-related activation convergence following cognitive training. Results: Cognitive training was associated with reliable behavioral improvement at the aggregate level (Hedges’ g = 0.546, 95% CI [0.414, 0.678]) and within both children/adolescents and young adults. SDM results indicated that task-related activation changes were spatially heterogeneous and included both increases and decreases across distributed brain regions. In age-stratified analyses, spatial convergence within children/adolescents involved occipital and frontoparietal regions; within young adults, it involved cingulo-insular and posterior midline regions. Exploratory behavioral moderator analyses indicated outcome-dependent variation, with smaller effects for transfer outcomes and larger effects in studies with greater training exposure. Conclusions: Behavioral improvements across included designs were observed in both age groups, whereas task-related activation convergence exhibited spatially heterogeneous, age-stratified patterns. These findings suggest that training-related neural effects are better characterized as spatial convergence patterns across studies rather than as a single uniform activation change. The age-stratified maps describe spatial convergence within each age group and should not be interpreted as statistically established developmental differences.

## 1. Introduction

Cognitive training offers a controlled way to examine how repeated, goal-directed practice relates to changes in cognitive performance and task-related brain activity [1,2,3]. It generally refers to structured interventions that repeatedly engage specific cognitive operations with the aim of modifying cognitive processing and improving behavioral performance [3,4,5]. Such interventions have been widely used in education, rehabilitation, and cognitive neuroscience, targeting domains such as working memory, attention, inhibitory control, and cognitive flexibility [1,6,7]. The basic premise is that repeated practice can induce experience-dependent changes in cognitive systems and, under appropriate conditions, produce measurable behavioral gains [1,2,7]. However, the scope and interpretation of these gains remain debated, particularly with respect to whether cognitive training produces broad domain-general improvement or more selective optimization of trained processes and closely related task states [3,5,8,9].

A growing body of behavioral evidence favors a selective account of cognitive training effects rather than a broad domain-general account [8,9,10,11]. Gains are typically largest for trained tasks and near-transfer outcomes, whereas far-transfer effects and broad improvements in general cognition are smaller and less consistent [5,9,11]. This pattern suggests that training effects depend strongly on the overlap between the trained operation and the outcome measure, including task rules, stimulus–response mappings, and representational demands [3,4,12]. Individual differences, including baseline ability and developmental stage, further shape whether and how training effects generalize beyond the practiced task [3,4,13]. Cognitive training is therefore better understood as a set of experience-dependent interventions, the effects of which vary according to the alignment of training content, assessment demands, and participant characteristics.

This selectivity is especially important in developmental populations. Childhood and adolescence are periods of ongoing maturation in executive functions, attentional control, and higher-order cognitive regulation [14,15,16]. In these age groups, training-related improvement is unlikely to depend only on the specific cognitive operation being trained. It is also likely to depend on the developmental state of the systems supporting task engagement, strategy use, and transfer [15,17,18]. Empirical reviews indicate that children and adolescents can benefit from cognitive training, but the size and generality of these effects vary across task demands, intervention formats, and developmental readiness [19,20,21,22,23]. In developmental samples, cognitive training effects should therefore be interpreted in relation to both the content of training and the maturational context in which training takes place.

Task-based neuroimaging provides a complementary approach for examining whether behavioral improvement following cognitive training is accompanied by changes in task-related brain activation [24,25]. Evidence from practice and training studies shows that repeated task engagement can alter the functional organization supporting performance, including redistribution of activation, increased recruitment of task-relevant regions, and reduced engagement of control-related regions as performance becomes more efficient [24,25,26,27,28]. Findings from cognitive training studies, however, have been highly heterogeneous. Some studies report increased activation in control, parietal, sensory, or cerebellar regions, whereas others report decreased activation or no significant whole-brain changes [29,30,31]. These mixed findings indicate that training-related neural changes are unlikely to follow a single direction or mechanism. Instead, they may involve different combinations of strategy change, altered task demands, attentional allocation, control load, and processing efficiency [3,29,30,31].

One way to organize this heterogeneity is to distinguish between activation increases and decreases without assuming that either pattern has a fixed meaning. Increased post-training activation can be interpreted as stronger recruitment of task-relevant regions or compensatory engagement of auxiliary systems, depending on the task and population [24,26,32]. Decreased activation, by contrast, is often discussed in relation to improved processing efficiency, reduced control demands, or automatization [33]. Training may also change the balance between task-specific recruitment and broader control-related engagement, particularly in networks involved in salience processing, cognitive control, and task-set regulation [34,35,36]. Age is a plausible source of variation in these patterns, although age differences in the literature may also reflect differences in training paradigms, task composition, and study design [15,17,18]. During childhood and adolescence, frontoparietal and cingulo-opercular systems continue to undergo differentiation, integration, and functional specialization [15,16,37]. Training-related improvement during this period may therefore involve greater task-related recruitment or stabilization of developing control systems. In young adults, where task networks are generally more mature, improvement may be more closely related to optimization of existing processing profiles and reduced control-related engagement [2,33,38]. Thus, age-stratified analyses may help characterize task-related activation patterns separately within children/adolescents and young adults.

Despite the theoretical relevance of this issue, age-stratified patterns of cognitive training-related neural effects have not been systematically synthesized. Previous work has primarily focused on behavioral transfer, specific training domains, or narrative summaries of neuroimaging findings [1,5,9,30,31]. It remains unclear whether the neuroimaging literature shows reproducible patterns of task-related activation convergence following cognitive training, whether these patterns can be characterized separately within children/adolescents and young adults, and whether behavioral or intervention characteristics account for variability in neural outcomes. Coordinate-based meta-analytic methods are well suited to address these questions because they identify spatial convergence across heterogeneous neuroimaging studies while accommodating differences in task paradigms and experimental designs [39,40]. Therefore, the present study integrated a three-level behavioral meta-analysis with a Seed-based Mapping (SDM) coordinate-based meta-analysis to examine behavioral improvements and characterize age-stratified patterns of spatial convergence in task-related brain activation following cognitive training.

## 2. Materials and Methods

We conducted an SDM meta-analysis to synthesize task-related brain activation changes following cognitive training, following established recommendations for neuroimaging meta-analyses [39,40] and SDM procedures [41,42,43,44]. The review was reported in accordance with PRISMA 2020 guidelines [45], and the protocol was prospectively registered in PROSPERO (CRD42024599205). The completed PRISMA checklist is provided in Supplementary Table S1.

### 2.1. Inclusion and Exclusion Criteria

We included studies of children/adolescents (<18 years) and young adults (18–35 years) that used structured cognitive training interventions targeting working memory, attention, inhibitory control, executive function, or related cognitive processes. Neuroimaging studies were eligible if they reported whole-brain task-related activation changes using peak coordinates or explicitly reported null whole-brain findings. Behavioral eligibility was assessed separately within this neuroimaging-eligible set. When an eligible neuroimaging study reported extractable in-scan task-performance data, these data were coded for behavioral meta-analysis; studies without extractable behavioral data were retained in the SDM analysis but were not included in the behavioral meta-analysis. Given the limited task-fMRI evidence base, eligible designs included within-subject pre–post comparisons, between-group comparisons, and group-by-time or condition-by-time interaction designs, consistent with prior coordinate-based meta-analyses of training-related neural effects [46,47,48,49,50,51,52,53]. Region-of-interest-only studies and studies without whole-brain neuroimaging data were excluded, in line with standards for coordinate-based meta-analysis [39].

### 2.2. Literature Search Strategy

We searched PubMed, MEDLINE, PsycINFO, Embase, and Web of Science from database inception to 12 November 2024, using search terms related to cognitive training and task-based neuroimaging. Reference lists of included studies and relevant reviews were additionally screened through 20 June 2026 to identify eligible records. Study screening and data extraction were performed independently by two authors (TF and CL). Disagreements were resolved through discussion. Across study-screening decisions and duplicate data-extraction fields, the overall agreement rate between TF and CL before consensus was 97%. For each screening decision or extraction field, agreement was coded as 1 and disagreement as 0 according to the coding protocol; the agreement rate was the sum of agreements divided by the total number of duplicate decisions and fields.

Study-level information included sample characteristics, intervention features, control conditions, and task paradigms. Intervention characteristics included training approach (process- vs. strategy-based), adaptivity (adaptive vs. non-adaptive), and training dose, defined by the number of sessions and total training duration. For neuroimaging outcomes, we extracted peak coordinates, coordinate space, statistical values, task type, and contrast information. Studies reporting null whole-brain findings were included as empty peak files to reduce bias from selectively synthesizing only positive coordinate findings. Database-specific search strategies are reported in Supplementary Table S2, detailed study characteristics and final reporting-quality scores are provided in Supplementary Table S3, and the reporting-quality checklist and item-level ratings are provided in Supplementary Tables S4 and S5.

### 2.3. Behavioral Meta-Analysis

Behavioral effects were summarized as Hedges’ g [54], with positive values indicating improvement. For controlled studies, g was calculated from the standardized difference between the training and control groups, using post-intervention scores when available and pre–post change scores otherwise. For single-group pre–post studies, g was calculated from the standardized pre–post change. For formula-derived single-group pre–post effects, a pre–post correlation of r = 0 was used to estimate the sampling variance when this correlation was unavailable. Small-sample correction was applied to all effect sizes. All eligible outcomes and comparisons were retained as separate effect sizes. Because several studies contributed more than one effect size, we used three-level random-effects meta-analytic models to account for dependency among effect sizes nested within studies [55,56]. Models were estimated using restricted maximum likelihood. Residual heterogeneity was quantified using Q_E and variance components at the between- and within-study levels; corresponding I^2^ values were calculated using a typical sampling variance. The primary model estimated pooled behavioral improvement across all included outcomes. Moderator analyses examined whether behavioral effects varied by training approach, adaptivity, task-transfer status, training dose, study design, and sex composition. Analyses were conducted in R (version 4.6.0) using the metafor package (version 5.0-1).

### 2.4. SDM Meta-Analysis

Coordinate-based neuroimaging meta-analyses were performed using SDM, version 5.15 [41,42,43,44]. All coordinates were converted to Montreal Neurological Institute (MNI) space. A 20 mm full-width-at-half-maximum isotropic Gaussian kernel was used to recreate study-level activation maps, and random-effects analyses were conducted for the full sample and for the age-stratified samples. The main SDM analyses identified cross-study spatial convergence of directionally coded, training-related task-activation effects following cognitive training. For within-group pre–post contrasts, positive and negative directions represented post-training increases and decreases, respectively. For controlled between-group or interaction contrasts, positive and negative directions represented greater and lower activation, respectively, in the training condition relative to the comparator. Following prior SDM coordinate-based meta-analyses [39,44,46,47,48,49,50,51,52,53], the primary exploratory maps were estimated using a conventional uncorrected threshold of voxel-wise *p* < 0.005, absolute SDM-Z > 1, and cluster extent k ≥ 100 voxels. This threshold was used to facilitate the exploratory identification of spatial convergence across the heterogeneous and limited task-fMRI literature. Exploratory SDM meta-regression analyses examined whether intervention and task characteristics were associated with variability in activation patterns. The moderators included training approach, adaptivity, task-transfer status, behavioral improvement, training dose, study design, and sex composition. Behavioral effect sizes were entered as study-level exploratory moderators. These analyses were interpreted as evidence of spatial convergence across studies rather than as direct within-study brain-behavior coupling. Age-stratified SDM analyses were conducted to descriptively characterize spatial convergence within children/adolescents and young adults. As prespecified under “Planned Data Synthesis” in PROSPERO, only moderator clusters overlapping the corresponding significant main-effect regions were retained to reduce false-positive findings, consistent with prior studies [39,44,46,47,48,49,50,51,52,53]. The 137 eligible contrasts were entered separately in the primary SDM analysis. To assess within-sample dependence, we repeated the overall analysis after combining contrasts from the same sample using SDM’s Combine Images function (r = 0.50), yielding 77 sample-level maps.

### 2.5. Sensitivity and Reliability Analyses

The robustness of the behavioral findings was examined by varying the assumed pre–post correlation from r = 0 to r = 0.70. Small-study effects were assessed descriptively after aggregating dependent effects within each study using inverse-variance weights. The diagnostics included funnel plots, Egger regression [57], trim-and-fill procedures [58], and PEESE models [59,60]. These procedures were treated as complementary descriptive diagnostics rather than interchangeable corrections; no single procedure was treated as the principal basis for inference, which remained the three-level random-effects model. For the SDM analyses, robustness was assessed using leave-one-out jackknife analyses [39,44], with the meta-analysis rerun after omitting one study at a time. Detailed jackknife results are provided in Supplementary Tables S6–S8.

To determine whether the principal findings were driven by study design, the behavioral meta-analysis and SDM analysis were repeated using only within-group pre–post effects and contrasts, respectively. The primary young-adult age range was 18–35 years. As a boundary sensitivity analysis, we repeated the age-stratified behavioral and SDM analyses after restricting the young-adult group to studies with mean ages of 18–30 years. This restriction excluded three behavioral studies and four SDM studies with mean participant ages that exceeded 30 years; the studies and reasons are identified in Supplementary Table S3.

### 2.6. Reporting-Quality Assessment

Reporting quality was independently assessed at the item level by TF and CL for all included studies using a project-specific adaptation of the 17-item neuroimaging reporting-quality checklist described by Lavallée et al. [61]. The checklist covered sampling, task design, imaging acquisition, preprocessing, and statistical reporting; each item was scored 0, 0.5, or 1, yielding a total score from 0 to 17. Disagreements were resolved through discussion. Reporting quality was examined as a study-level moderator in behavioral sensitivity analyses and was not used as a basis for study exclusion. This instrument assesses reporting completeness and technical methodological features rather than formal risk of bias; no separate formal risk-of-bias assessment was undertaken. The checklist items and study-level item ratings are reported in Supplementary Tables S4 and S5, respectively, and final reporting-quality scores are included in Supplementary Table S3.

## 3. Results

### 3.1. Study Selection and Study Characteristics

**Figure 1:**
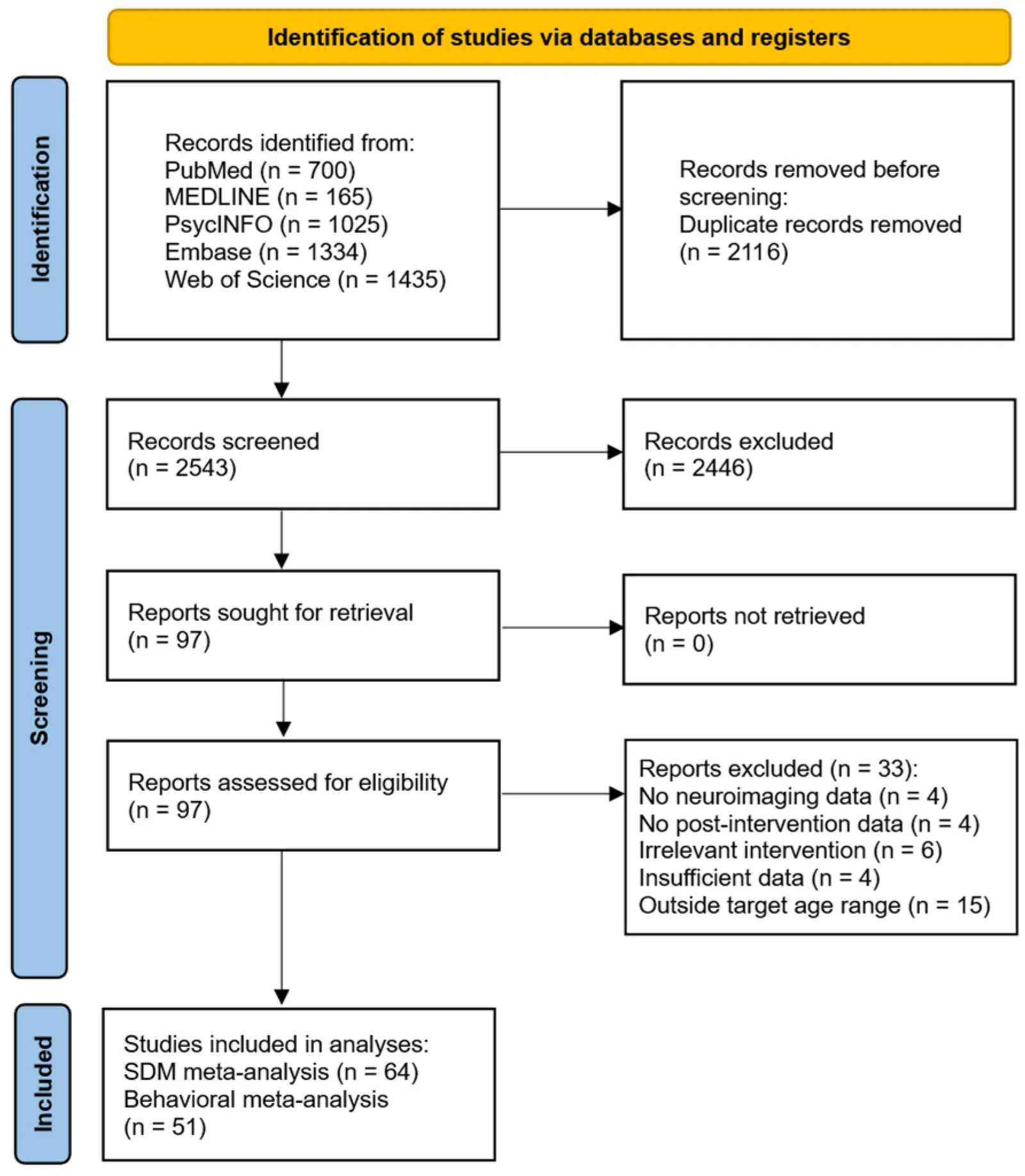
PRISMA flowchart detailing the process for screening and including relevant studies. *Note.* The PRISMA 2020 flow diagram summarizes database records identified from PubMed, MEDLINE, PsycINFO, Embase, and Web of Science searches through 12 November 2024, plus supplementary citation searching through 20 June 2026. Numbers indicate records or reports at each screening stage before the final behavioral and SDM analytic samples were defined.

The study selection process is summarized in Figure 1. Children/adolescents were defined as participants younger than 18 years, whereas young adults were defined as individuals aged 18–35 years for the age-stratified analyses. After duplicates were removed, 2543 records were screened by title and abstract, and 97 reports were assessed in full text. In total, 64 studies met the inclusion criteria for the SDM coordinate-based meta-analysis, contributing 137 eligible functional neuroimaging contrasts [62,63,64,65,66,67,68,69,70,71,72,73,74,75,76,77,78,79,80,81,82,83,84,85,86,87,88,89,90,91,92,93,94,95,96,97,98,99,100,101,102,103,104,105,106,107,108,109,110,111,112,113,114,115,116,117,118,119,120,121,122,123,124,125]. The behavioral meta-analysis included 51 of these studies, contributing 205 effect sizes (summed N = 1838) [62,63,64,65,66,67,68,69,70,71,72,73,74,75,76,77,78,79,80,81,82,83,84,85,86,87,88,89,90,91,92,93,94,95,96,97,98,99,100,101,102,103,104,105,106,107,108,109,110,111,112]. Six studies reported null whole-brain findings [110,111,112,113,114,115]. Among the included studies, 20 focused on children/adolescents and 45 on young adults; one study [110] reported separate samples in both age groups.

Across the neuroimaging evidence, the summed sample size was 2416. Child/adolescent and young-adult samples accounted for 28.8% and 71.3% of the neuroimaging data, respectively. Mean ages ranged from 7.75 to 16.62 years in the child/adolescent group and from 20.80 to 31.50 years in the young-adult group. Most interventions were process-based (90.0%), and 61.3% used adaptive training. Working-memory paradigms were the most common in-scan tasks (48.8%), and controlled/interaction contrasts accounted for 54.7% of the eligible neuroimaging contrasts. Detailed information on the included studies is provided in Supplementary Table S3. Database-specific search strategies, reasons for exclusion, and full coding information are provided in the Supplementary Materials.

### 3.2. Behavioral Meta-Analysis

#### 3.2.1. Pooled Behavioral Improvement Across Included Designs

**Figure 2:**
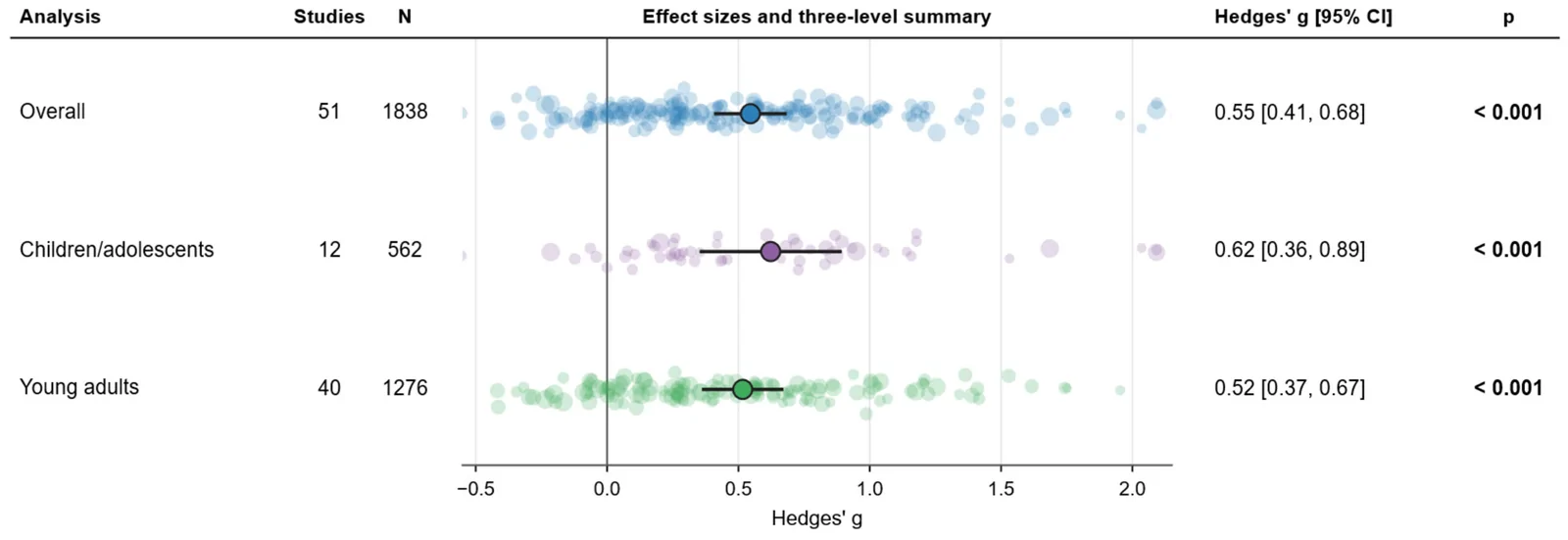
Forest plot for pooled behavioral improvement across included designs. *Note.* Effects are Hedges’ g from the three-level random-effects behavioral meta-analysis, with dependent effect sizes nested within studies and restricted maximum likelihood estimation. Points indicate pooled estimates, horizontal lines indicate 95% confidence intervals, positive values indicate behavioral improvement across included designs, and N indicates the summed participant count.

The three-level behavioral meta-analysis indicated pooled behavioral improvement across the included designs (205 effect sizes from 51 studies; Hedges’ g = 0.546; SE = 0.067; 95% CI [0.414, 0.678]; z = 8.103; *p* < 0.001). Significant pooled effects were also observed in children/adolescents (58 effect sizes from 12 studies; Hedges’ g = 0.623; SE = 0.135; 95% CI [0.359, 0.887]; z = 4.624; *p* < 0.001) and young adults (147 effect sizes from 40 studies; Hedges’ g = 0.516; SE = 0.076; 95% CI [0.367, 0.665]; z = 6.797; *p* < 0.001; Figure 2). Residual heterogeneity was substantial overall (τ^2^between = 0.146; τ^2^within = 0.113; I^2^ = 69.6%; Q_E(204) = 678.45; *p* < 0.001).

#### 3.2.2. Exploratory Behavioral Moderator Analyses

**Figure 3:**
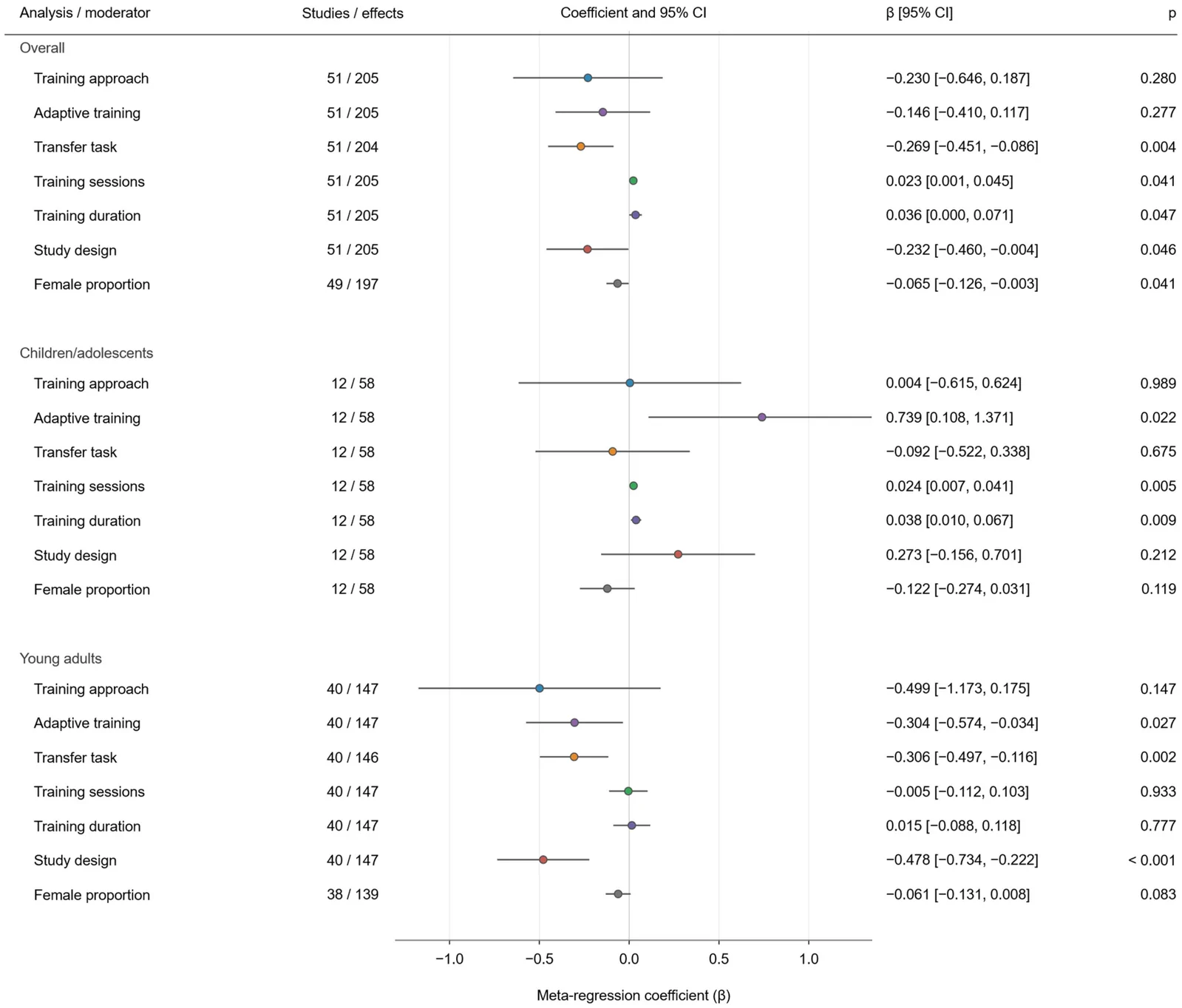
Behavioral moderator analyses. *Note.* Points indicate single-moderator meta-regression coefficients (β), and horizontal lines indicate 95% CIs. Study/effect counts are shown for each analysis; counts for female proportion reflect the studies with available sex-composition data. Binary moderators were coded as 1 versus 0: process- versus strategy-based training, adaptive versus non-adaptive training, transfer versus trained/same-task outcomes, and controlled/interaction versus within-group pre–post designs. For presentation only, coefficients for continuous moderators are scaled per 10 training sessions, per 1000 min of training duration, and per 10-percentage-point increase in female proportion; this scaling does not change the corresponding test statistics or *p*-values. Estimates were obtained from three-level random-effects models accounting for dependent effect sizes within studies.

Exploratory three-level moderator analyses identified several candidate sources of variation in behavioral improvement (Figure 3). In the overall sample, transfer outcomes showed smaller effects than trained or same-task outcomes (β = −0.269; SE = 0.093; 95% CI [−0.451, −0.086]; z = −2.889; *p* = 0.004), whereas a greater number of training sessions was associated with larger behavioral improvement (β = 0.002; SE = 0.001; 95% CI [0.000, 0.005]; z = 2.047; *p* = 0.041). Longer total training duration was also associated with larger improvement (β = 0.036 per 1000 min; SE = 0.018; 95% CI [0.000, 0.071]; z = 1.985; *p* = 0.047), whereas a higher proportion of female participants was associated with smaller improvement (β = −0.065 per 10 percentage points; SE = 0.032; 95% CI [−0.126, −0.003]; z = −2.047; *p* = 0.041). Controlled/interaction designs also showed smaller effects than within-group pre–post designs (β = −0.232; SE = 0.116; 95% CI [−0.460, −0.004]; z = −1.994; *p* = 0.046).

In children/adolescents, larger behavioral improvement was observed in studies using adaptive training (β = 0.739; SE = 0.322; 95% CI [0.108, 1.371]; z = 2.294; *p* = 0.022) and in studies with a greater number of training sessions (β = 0.002; SE = 0.001; 95% CI [0.001, 0.004]; z = 2.816; *p* = 0.005) or longer total training duration (β = 0.038 per 1000 min; SE = 0.015; 95% CI [0.010, 0.067]; z = 2.625; *p* = 0.009). In young adults, smaller behavioral improvement was observed for adaptive training (β = −0.304; SE = 0.138; 95% CI [−0.574, −0.034]; z = −2.209; *p* = 0.027), transfer outcomes (β = −0.306; SE = 0.097; 95% CI [−0.497, −0.116]; z = −3.149; *p* = 0.002), and controlled/interaction designs relative to within-group pre–post designs (β = −0.478; SE = 0.131; 95% CI [−0.734, −0.222]; z = −3.654; *p* < 0.001).

### 3.3. SDM Coordinate-Based Meta-Analysis

#### 3.3.1. Main Task-Related Activation Changes

**Figure 4:**
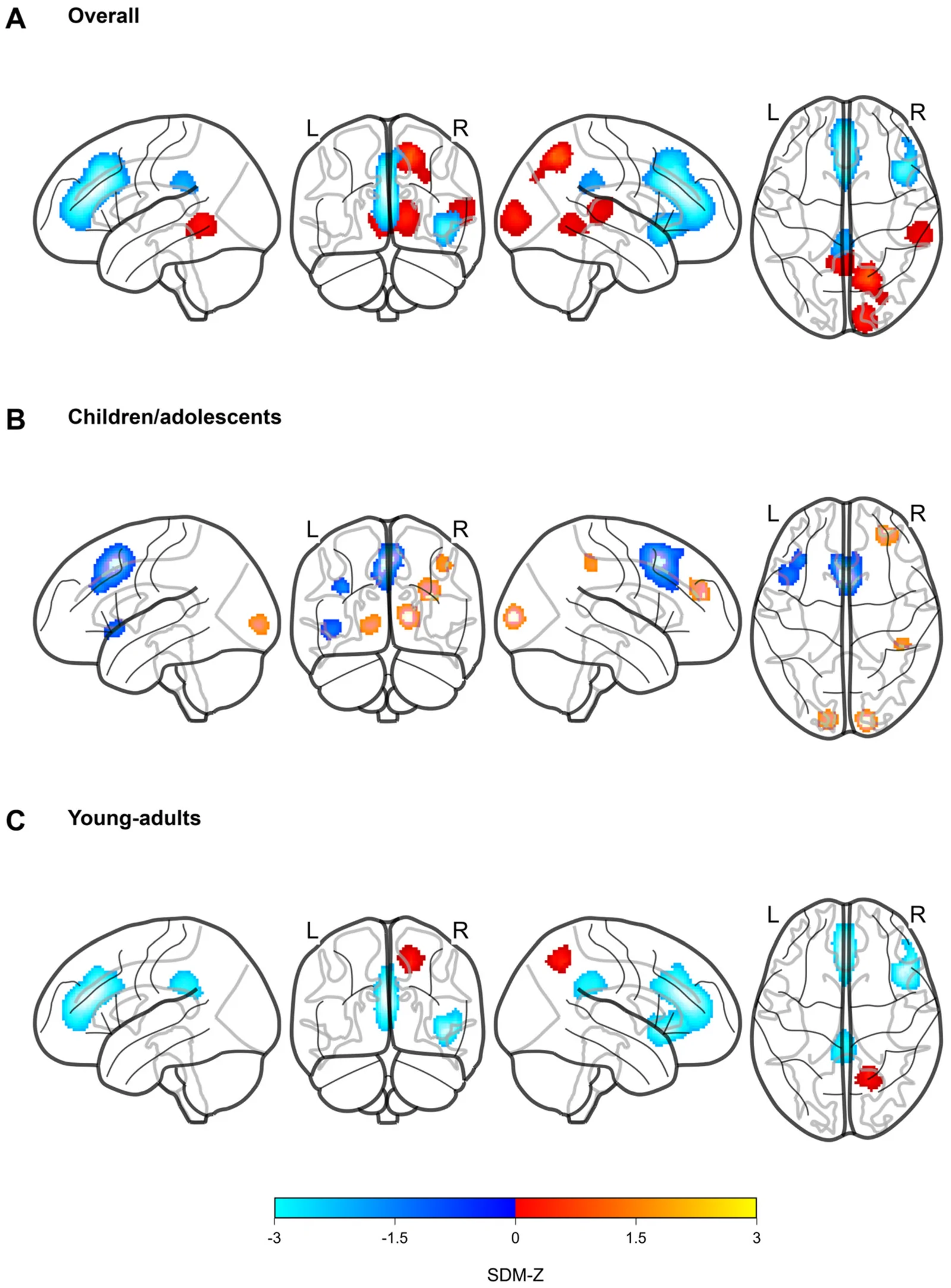
Main SDM result maps on a common color scale: (**A**) overall sample; (**B**) children/adolescents; (**C**) young adults. *Note.* Maps show SDM 5.15 random-effects coordinate-based meta-analytic results in MNI space. All panels use the same SDM-Z color scale (−3 to 3). Main-analysis maps used a 20 mm full-width-at-half-maximum kernel and were thresholded at voxel-wise *p* < 0.005, absolute SDM-Z > 1, and cluster extent k ≥ 100 voxels.

The overall SDM analysis revealed seven significant clusters of task-related activation change following cognitive training (Table 1; Figure 4). Clusters showing increased activation were located in the right superior parietal lobule (R SPL), right occipital pole/primary visual cortex (R OP/V1), left cerebellar lobule IV/V, and a region adjacent to the right posterior superior temporal gyrus (R posterior STG). Clusters showing decreased activation were located in the left anterior cingulate/paracingulate cortex (L ACC/paracingulate), right insula, and left posterior cingulate cortex (L PCC).

In children/adolescents, seven significant clusters were detected. Increased activation was found in the right cuneus, right middle frontal gyrus (R MFG), L OP/V1, and right inferior parietal lobule (R IPL), whereas decreased activation was found in the left supplementary motor area (L SMA), a region adjacent to L MFG, and the left insula. In young adults, four significant clusters emerged: increased activation in the R SPL/precuneus region and decreased activation in L ACC/paracingulate, right insula, and L PCC.

#### 3.3.2. Exploratory SDM Moderator Effects

In the overall sample, task-transfer status was associated with a cluster overlapping the L ACC/paracingulate main-effect region, with the peak located in the R SMA (MNI = 8, 22, 54; SDM-Z = 2.111; *p* < 0.001; k = 1345).

In children/adolescents, behavioral improvement was associated with a cluster overlapping the R MFG main-effect region (MNI = 30, 50, and 30; SDM-Z = 1.050; *p* < 0.001; k = 318). Additional moderator effects involved task-transfer status in bilateral OP/V1 and the R IPL, training approach in bilateral OP/V1, training adaptivity in the right cuneus and a region adjacent to the L MFG, and both training sessions and total training duration in the left middle occipital gyrus (L MOG).

In young adults, behavioral improvement was associated with a cluster overlapping the right angular gyrus (R AG). Task-transfer status overlapped the L ACC/paracingulate main-effect region, with the peak located in the right medial superior frontal gyrus (R medial SFG). Training adaptivity was associated with clusters in the right precuneus, L PCC, and right inferior frontal gyrus pars opercularis (R IFG opercularis). Training sessions were associated with clusters in the R SMA and left precuneus, whereas the proportion of female participants was associated with clusters in the L PCC and R IFG triangularis. The moderator results are summarized in Table 2. No study-design SDM moderator cluster met the prespecified main-effect overlap criterion.

### 3.4. Reliability and Sensitivity Analyses

The behavioral findings were robust to alternative assumptions about the pre–post correlation. Across correlation values from r = 0 to r = 0.70, the overall pooled estimate remained significant (Hedges’ g range = 0.541–0.546; all *p* < 0.001). The effects also remained significant in children/adolescents (Hedges’ g range = 0.609–0.623; all *p* < 0.001) and young adults (Hedges’ g range = 0.515–0.519; all *p* < 0.001; Supplementary Table S9).

Study-level small-study-effect diagnostics did not provide consistent evidence of publication bias in the behavioral meta-analysis. Egger tests were not significant for the overall sample (*p* = 0.751), children/adolescents (*p* = 0.401), or young adults (*p* = 0.228). Trim-and-fill analyses imputed no missing studies (k0 = 0), and PEESE-adjusted estimates remained positive across all groups. These complementary diagnostics were interpreted descriptively; no individual procedure was treated as decisive. Complete results are reported in Supplementary Table S10 and Supplementary Figure S1.

Cluster-level SDM funnel and Egger diagnostics suggested that asymmetry varied across clusters. In the overall sample, 4 of 7 main-analysis clusters showed *p* < 0.05, whereas no cluster-level asymmetry was detected in children/adolescents. In young adults, 3 of 4 main-analysis clusters showed *p* < 0.05. Full SDM funnel and Egger diagnostics are reported in Supplementary Table S11 and Supplementary Figure S2.

The sample-level analysis retained all seven clusters identified in the primary overall analysis, with substantial spatial overlap between the two maps (Dice = 0.748; Supplementary Table S12).

Restricting the behavioral meta-analysis to within-group pre–post effects continued to show significant behavioral improvement (g = 0.666; SE = 0.090; 95% CI [0.490, 0.843]; *p* < 0.001). Similarly, the SDM analysis restricted to within-group pre–post contrasts retained five of the seven spatial patterns identified in the overall analysis, involving the R SPL, L cerebellar lobule IV/V, R posterior STG, L ACC/paracingulate, and R insula; the OP/V1 and PCC patterns were not retained.

Reporting-quality sensitivity analyses did not identify reliable associations in the primary 18–35-year dataset for the overall sample (*p* = 0.678), children/adolescents (*p* = 0.307), or young adults (*p* = 0.145).

Restricting the young-adult upper age boundary to 30 years did not materially alter the primary behavioral meta-analytic estimate or the overall pattern of spatial convergence in the age-stratified SDM analyses. These findings indicate that the primary results were not dependent on the 18–35-year upper age boundary.

Leave-one-out jackknife analyses indicated that the main SDM clusters were not driven by any single study. Across the overall, children/adolescents, and young-adult analyses, the main clusters remained stable following the sequential omission of individual studies (Supplementary Tables S6–S8).

## 4. Discussion

By combining a three-level behavioral meta-analysis with coordinate-based synthesis of task-based neuroimaging evidence, this study examined behavioral improvement and task-related activation changes following cognitive training in children/adolescents and young adults. Three main observations emerged. First, cognitive training was linked to reliable behavioral improvement across the included outcomes, and this effect was present in both children/adolescents and young adults. Second, the neuroimaging findings did not support a single direction of activation change. Instead, task-related activation convergence was spatially distributed and included both increases and decreases. Third, the age-stratified analyses described spatial convergence profiles separately within children/adolescents and young adults.

At the behavioral level, the results indicate measurable pooled improvement across a broad set of outcomes, including trained-task, near-transfer, transfer, and standardized measures. Because the evidence base included both controlled/interaction and within-group pre–post designs, this overall estimate should not be interpreted as a uniform causal effect or as evidence for unrestricted general cognitive enhancement. Rather, it fits with prior work showing that training-related improvement is often largest when the trained task and the outcome measure share cognitive operations, task rules, or representational demands [5,9,12,126]. This selective interpretation is also consistent with the limited and low-certainty evidence for broad cognitive benefits from computerized training in cognitively healthy midlife populations [127]. Thus, the present findings support a selective pattern of behavioral improvement, the magnitude of which depends on the match between intervention content and assessment demands.

At the neural level, the SDM results revealed a heterogeneous pattern of activation convergence across distributed regions. Increased convergence was found in parietal, occipital, and cerebellar regions, whereas decreased convergence was found in cingulo-insular and posterior midline regions. This mixture of increases and decreases is consistent with prior practice and training studies showing that repeated task engagement can be accompanied by different activation patterns depending on task demands, strategy use, and stage of learning [24,26,29,30]. Increases in task-related activation may indicate stronger recruitment of task-relevant or compensatory processing systems [24,32], whereas decreases may indicate reduced control demands, greater processing efficiency, or task familiarization [33,35]. A broader healthy-population neuroimaging synthesis likewise supports distributed training-related changes in task recruitment rather than a single localized mechanism [46]. Functional-network evidence from vascular cognitive impairment further suggests that training may be accompanied by distributed changes in task recruitment and network organization [128]. These interpretations should remain cautious, however, because coordinate-based meta-analysis identifies spatial convergence across reported peaks rather than directly testing the neural mechanisms underlying training-related change.

The present results also extend, while not duplicating, prior working-memory neuroimaging meta-analyses by Vartanian et al. and Zhang et al. [30,31]. Those studies focused specifically on working-memory training and highlighted distributed changes in task-relevant control and frontoparietal systems. By including a broader range of cognitive-training paradigms and age groups, the present synthesis also identified parietal, occipital, cerebellar, cingulo-insular, and posterior-midline convergence. This partial overlap and divergence may reflect neuroplastic changes in task recruitment, strategy selection, and processing efficiency, but such mechanisms remain plausible interpretations rather than direct evidence from the coordinate-based findings.

When the analyses were stratified by age group, spatial convergence in children/adolescents included increases in occipital and frontoparietal regions, together with decreases in SMA, frontal, and insular regions. In young adults, convergence included decreases in cingulo-insular and posterior midline regions, alongside increases in the R SPL/precuneus region. These findings characterize patterns within the respective age-stratified evidence bases and may also reflect differences in task paradigms, training content, and intervention design across studies [30].

Several limitations should be noted. First, coordinate-based meta-analysis summarizes the spatial convergence of reported peaks and cannot recover full statistical maps, which limits mechanistic interpretation [39,43,129]. Second, the included studies varied substantially in training protocols, control conditions, behavioral outcomes, scanned tasks, and contrast designs, which may have jointly contributed to the distributed activation patterns observed here [30]. Differences in the number of studies and statistical power across age strata may also have influenced apparent differences in spatial convergence. Third, the exploratory moderator analyses were based on reduced effective sample sizes and should be interpreted as hypothesis-generating rather than confirmatory evidence. In addition, given the heterogeneous and limited task-fMRI evidence base and the use of an uncorrected primary SDM threshold consistent with prior coordinate-based meta-analyses [39,44,46,47,48,49,50,51,52,53], the reported SDM clusters should be interpreted as exploratory patterns of spatial convergence rather than as definitive neural effects. The cluster-level SDM asymmetry signals in part of the overall and young-adult maps provide an additional reason for caution. Fourth, behavioral and neuroimaging outcomes were not always aligned at the same analytic level. As a result, the present study can describe how behavioral improvement and activation convergence co-occur across the literature, but it cannot establish direct brain-behavior relationships within individual studies. Finally, the age-stratified findings should be interpreted as descriptive patterns within each age group rather than as statistically established developmental differences. The project-specific checklist evaluated reporting quality rather than formal risk of bias, which limits conclusions about bias within the primary studies.

## 5. Conclusions

Cognitive training was associated with reliable behavioral improvement within both children/adolescents and young adults. Task-related activation convergence was spatially heterogeneous within the age-stratified evidence bases. Rather than indicating a single directional neural effect, the findings suggest that training-related activation changes are better understood as distributed spatial convergence patterns observed within each age group. The behavioral moderator results further indicate outcome-dependent variation in training effects, particularly for transfer outcomes and training exposure. Overall, this study indicates that behavioral gains and task-related activation changes co-occur across the literature following cognitive training, while also highlighting task heterogeneity when interpreting coordinate-based neuroimaging evidence. Future studies using harmonized task designs, longitudinal protocols, and more closely aligned behavioral and neuroimaging outcomes are needed to clarify how training design and participant characteristics shape cognitive and neural change.

## Figures and Tables

**Table 1:** Brain regions showing significant task-related activation changes after cognitive training.

MNI Coordinate	SDM-Z	*p*	Voxels	Description
*Overall*				
18,−64,54	1.522	<0.001	771	R SPL
14,−94,6	1.222	<0.001	769	R OP/V1
−6,−50,0	1.131	<0.001	639	L cerebellum lobule IV/V
60,−32,10	1.061	<0.001	562	R posterior STG
−2,28,28	−2.930	<0.001	3054	L ACC/paracingulate
44,20,−2	−2.484	<0.001	989	R insula
−2,−34,30	−1.888	0.002	330	L PCC
*Children/adolescents*				
16,−96,6	1.685	<0.001	386	R cuneus
32,50,22	1.601	<0.001	327	R MFG
−14,−92,0	1.487	<0.001	254	L OP/V1
44,−34,46	1.354	0.002	117	R IPL
0,18,48	−1.668	<0.001	1567	L SMA
−34,28,30	−1.259	<0.001	137	L MFG
−42,16,−4	−1.154	<0.001	257	L insula
*Young−adult*				
18,−56,50	1.209	<0.001	382	R SPL/precuneus
−2,34,24	−2.919	<0.001	1981	L ACC/paracingulate
46,22,−2	−2.800	<0.001	967	R insula
−4,−38,30	−2.295	<0.001	603	L PCC

*Note.* Brain regions showing task-related activation effects from the random-effects SDM meta-analysis. The sign of SDM-Z indicates direction. Threshold: voxel-wise *p* < 0.005, |SDM-Z| > 1, cluster extent ≥ 100 voxels (MNI space). The *p* values are permutation-derived SDM values and therefore do not correspond directly to a normal-theory transformation of SDM-Z. Abbreviations: L/R = left/right; ACC = anterior cingulate cortex; IPL = inferior parietal lobule; MFG = middle frontal gyrus; OP = occipital pole; PCC = posterior cingulate cortex; SMA = supplementary motor area; SPL = superior parietal lobule; STG = superior temporal gyrus; V1 = primary visual cortex.

**Table 2:** Brain regions showing significant moderator effects overlapping main-effect regions.

Moderator	MNI Coordinate	SDM-Z	*p*	Voxels	Description
*Overall*					
Transfer task	8,22,54	2.111	<0.001	1345	R SMA
*Children/adolescents*					
Behavioral improvement (Hedges’ g)	30,50,30	1.050	<0.001	318	R MFG
Transfer task	−14,−92,0	1.949	<0.001	213	L OP/V1
	18,−94,6	2.103	<0.001	292	R OP/V1
	42,−46,48	2.231	<0.001	400	R IPL
Training approach	18,−92,6	−2.710	<0.001	412	R OP/V1
	−16,−94,0	−2.446	<0.001	314	L MOG
Adaptive training	16,−96,6	1.491	<0.001	351	R cuneus
	−36,38,30	−1.437	<0.001	497	L MFG
Training sessions	−16,−94,0	4.826	<0.001	337	L MOG
Training duration	−16,−94,0	4.677	<0.001	323	L MOG
*Young-adult*					
Behavioral improvement (Hedges’ g)	34,−66,46	2.058	<0.001	615	R AG
Transfer task	6,34,50	1.875	<0.001	1001	R medial SFG
Adaptive training	6,−54,58	1.004	<0.001	404	R precuneus
	−8,−52,32	−2.208	<0.001	754	L PCC
	46,14,32	−2.135	<0.001	657	R IFG opercularis
Training sessions	4,16,50	2.502	<0.001	1127	R SMA
	−10,−54,30	−1.067	<0.001	338	L precuneus
Female participants (%)	−2,−52,22	−2.667	<0.001	917	L PCC
	52,26,8	−2.279	0.002	167	R IFG triangularis

*Note.* Moderator clusters were reported according to the prespecified spatial-overlap criterion described in Section 2.4. This interpretive anchoring rule relates exploratory moderator findings to the primary SDM maps but limits the range of reported moderator effects. Binary moderators were coded as 1 vs. 0 (transfer vs. trained/same task; process-based vs. strategy-based training; adaptive vs. non-adaptive training). Continuous moderators included behavioral improvement (Hedges’ g), number of training sessions, total training duration, and proportion of female participants. Threshold: voxel-wise *p* < 0.005, |SDM-Z| > 1, cluster extent ≥ 100 voxels (MNI space). Abbreviations: L/R = left/right; AG = angular gyrus; ACC = anterior cingulate cortex; IFG = inferior frontal gyrus; IPL = inferior parietal lobule; MFG = middle frontal gyrus; MOG = middle occipital gyrus; OP = occipital pole; PCC = posterior cingulate cortex; SFG = superior frontal gyrus; SMA = supplementary motor area; SPL = superior parietal lobule; STG = superior temporal gyrus; V1 = primary visual cortex.

## Data Availability

The data summarized in this review are provided in the article and Supplementary Materials. Additional analytic files are available from the corresponding author upon reasonable request.

## Supplementary Materials

The following supporting information can be downloaded at https://www.mdpi.com/article/10.3390/brainsci16080861/s1. Table S1: PRISMA 2020 checklist; Table S2: Database-specific search strategies; Table S3: Characteristics of included studies; Table S4: Reporting-quality assessment checklist; Table S5: Item-level reporting-quality ratings for the included studies; Table S6: Jackknife analysis for the overall main SDM analysis; Table S7: Jackknife analysis for the children/adolescents main SDM analysis; Table S8: Jackknife analysis for the young-adult main SDM analysis; Table S9: Behavioral sensitivity analysis using alternative pre-post correlation assumptions; Table S10: Behavioral funnel-plot, small-study-effect, and correction checks; Table S11: SDM cluster-level funnel-plot and Egger diagnostics for main-analysis clusters; Table S12: Sample-level sensitivity analysis of dependent SDM contrasts; Figure S1: Contour-enhanced funnel plots for the behavioral meta-analysis; Figure S2: Cluster-level SDM funnel plots for significant main-analysis clusters.
