# Supplementary material for: Behavioral Improvement and Task-Related Brain Activation Following Cognitive Training in Children/Adolescents and Young Adults: A Three-Level Behavioral and Coordinate-Based Neuroimaging Meta-Analysis
Source: Brain Sci. 2026 Aug 14;16(8):861. doi: 10.3390/brainsci16080861 (PMC13511496; doi:10.3390/brainsci16080861)
Supplement: Supplementary file 1 [file brainsci-16-00861-s001.zip › brainsci-4454581-supplementary.pdf]

Supplementary Materials

Supplementary Tables

Supplementary Table S1. PRISMA 2020 checklist.

Section and Topic	Item #	Checklist item	Location where item is reported
TITLE		TITLE	
Title	1	Identify the report as a systematic review.	Manuscript title page (page 1)
ABSTRACT		ABSTRACT	
Abstract	2	See the PRISMA 2020 for Abstracts checklist.	Abstract (page 1)
INTRODUCTION		INTRODUCTION	
Rationale	3	Describe the rationale for the review in the context of existing knowledge.	Introduction (pages 2–3)
Objectives	4	Provide an explicit statement of the objective(s) or question(s) the review addresses.	End of Introduction (page 3)
METHODS		METHODS	
Eligibility criteria	5	Specify the inclusion and exclusion criteria for the review and how studies were grouped for the syntheses.	Section 2.1 (pages 3–4)
Information sources	6	Specify all databases, registers, websites, organisations, reference lists and other sources searched or consulted to identify studies. Specify the date when each source was last searched or consulted.	Section 2.2 (page 4); Supplementary Table S2
Search strategy	7	Present the full search strategies for all databases, registers and websites, including any filters and limits used.	Supplementary Table S2
Selection process	8	Specify the methods used to decide whether a study met the inclusion criteria of the review, including how many reviewers screened each record and each report retrieved, whether they worked independently, and if applicable, details of automation tools used in the process.	Section 2.2 (page 4)
Data collection process	9	Specify the methods used to collect data from reports, including how many reviewers collected data from each report, whether they worked independently, any processes for obtaining or confirming data from study investigators, and if applicable, details of automation tools used in the process.	Section 2.2 (page 4)

Section and Topic	Item #	Checklist item	Location where item is reported
Data items	10a	List and define all outcomes for which data were sought. Specify whether all results that were compatible with each outcome domain in each study were sought (e.g. for all measures, time points, analyses), and if not, the methods used to decide which results to collect.	Sections 2.2–2.4 (pages 4–5); Supplementary Table S3
Data items	10b	List and define all other variables for which data were sought (e.g. participant and intervention characteristics, funding sources). Describe any assumptions made about any missing or unclear information.	Section 2.2 (page 4); Supplementary Table S3
Study risk of bias assessment	11	Specify the methods used to assess risk of bias in the included studies, including details of the tool(s) used, how many reviewers assessed each study and whether they worked independently, and if applicable, details of automation tools used in the process.	Not performed. Reporting quality was assessed separately (Section 2.6; Supplementary Tables S3–S5).
Effect measures	12	Specify for each outcome the effect measure(s) (e.g. risk ratio, mean difference) used in the synthesis or presentation of results.	Section 2.3 (page 4)
Synthesis methods	13a	Describe the processes used to decide which studies were eligible for each synthesis (e.g. tabulating the study intervention characteristics and comparing against the planned groups for each synthesis (item #5)).	Sections 2.1–2.4 (pages 3–5)
Synthesis methods	13b	Describe any methods required to prepare the data for presentation or synthesis, such as handling of missing summary statistics, or data conversions.	Sections 2.3–2.4 (pages 4–5)
Synthesis methods	13c	Describe any methods used to tabulate or visually display results of individual studies and syntheses.	Sections 3.2–3.4 (pages 7–12); Tables 1–2 and Figures 2–4
Synthesis methods	13d	Describe any methods used to synthesize results and provide a rationale for the choice(s). If meta-analysis was performed, describe the model(s), method(s) to identify the presence and extent of statistical heterogeneity, and software package(s) used.	Sections 2.3–2.4 (pages 4–5)
Synthesis methods	13e	Describe any methods used to explore possible causes of heterogeneity among study results (e.g. subgroup analysis, meta-regression).	Sections 2.3–2.4 (pages 4–5)
Synthesis methods	13f	Describe any sensitivity analyses conducted to assess robustness of the synthesized results.	Section 2.5 (page 5)
Reporting bias assessment	14	Describe any methods used to assess risk of bias due to missing results in a synthesis (arising from reporting biases).	Sections 2.5 and 3.4 (pages 5 and 12); Supplementary Tables S10–S11
Certainty assessment	15	Describe any methods used to assess certainty (or confidence) in the body of evidence for an outcome.	NA (certainty assessment was not conducted)

Section and Topic	Item #	Checklist item	Location where item is reported
RESULTS		RESULTS	
Study selection	16a	Describe the results of the search and selection process, from the number of records identified in the search to the number of studies included in the review, ideally using a flow diagram.	Section 3.1 (page 6); Figure 1
Study selection	16b	Cite studies that might appear to meet the inclusion criteria, but which were excluded, and explain why they were excluded.	Section 3.1 and Figure 1 (page 6)
Study characteristics	17	Cite each included study and present its characteristics.	Section 3.1 (page 6); Supplementary Table S3
Risk of bias in studies	18	Present assessments of risk of bias for each included study.	Not performed; total and item-level reporting-quality ratings are provided separately in Supplementary Tables S3–S5.
Results of individual studies	19	For all outcomes, present, for each study: (a) summary statistics for each group (where appropriate) and (b) an effect estimate and its precision (e.g. confidence/credible interval), ideally using structured tables or plots.	Figure 2 (page 7); effect-level data available from the corresponding author
Results of syntheses	20a	For each synthesis, briefly summarise the characteristics and risk of bias among contributing studies.	Study characteristics and total and item-level reporting-quality ratings are summarized in Supplementary Tables S3–S5; formal risk of bias was not assessed.
Results of syntheses	20b	Present results of all statistical syntheses conducted. If meta-analysis was done, present for each the summary estimate and its precision (e.g. confidence/credible interval) and measures of statistical heterogeneity. If comparing groups, describe the direction of the effect.	Sections 3.2–3.4 (pages 7–12); Tables 1–2 and Figures 2–4
Results of syntheses	20c	Present results of all investigations of possible causes of heterogeneity among study results.	Sections 3.2.2 and 3.3.2 (pages 7–11)
Results of syntheses	20d	Present results of all sensitivity analyses conducted to assess the robustness of the synthesized results.	Section 3.4 (page 12); Supplementary Tables S6–S12
Reporting biases	21	Present assessments of risk of bias due to missing results (arising from reporting biases) for each synthesis assessed.	Section 3.4 (page 12); Supplementary Tables S10–S11 and Figures S1–S2
Certainty of evidence	22	Present assessments of certainty (or confidence) in the body of evidence for each outcome assessed.	NA (certainty assessment was not conducted)

Section and Topic	Item #	Checklist item	Location where item is reported
DISCUSSION		DISCUSSION	
Discussion	23a	Provide a general interpretation of the results in the context of other evidence.	Discussion (pages 12–14)
Discussion	23b	Discuss any limitations of the evidence included in the review.	Limitations (pages 13–14)
Discussion	23c	Discuss any limitations of the review processes used.	Limitations (pages 13–14)
Discussion	23d	Discuss implications of the results for practice, policy, and future research.	Discussion and Conclusions (pages 12–14)
OTHER INFOR-MATION		OTHER INFORMATION	
Registration and proto-col	24a	Provide registration information for the review, including register name and registration number, or state that the review was not registered.	Methods; PROSPERO (CRD42024599205)
Registration and proto-col	24b	Indicate where the review protocol can be accessed, or state that a protocol was not prepared.	Methods; PROSPERO (CRD42024599205)
Registration and proto-col	24c	Describe and explain any amendments to information provided at registration or in the protocol.	The age eligibility ranges used for the present developmental synthesis were refined during the review (children/ad-olescents <18 years; young adults 18–35 years).
Support	25	Describe sources of financial or non-financial support for the review, and the role of the funders or sponsors in the review.	Funding statement (page 14)
Competing interests	26	Declare any competing interests of review authors.	Conflicts of Interest statement (page 15)
Availability of data, code and other materi-als	27	Report which of the following are publicly available and where they can be found: template data collection forms; data extracted from included studies; data used for all analyses; analytic code; any other materials used in the review.	Data Availability Statement (page 14)

Note. The checklist follows PRISMA 2020 reporting items. Item locations refer to the main manuscript or Supplementary Materials; items not applicable to this meta-analysis are marked as NA.

Supplementary Table S2. Database-specific search strategies.

Database	Search strategy	Records
PubMed	("cognitive training"[tiab] OR "cognitive rehabilitation"[tiab] OR "cognitive therapy"[tiab] OR "neuropsychological therapy"[tiab] OR "cognitive intervention"[tiab] OR "executive function training"[tiab] OR "working memory training"[tiab] OR "memory training"[tiab] OR "attention training"[tiab] OR "processing speed training"[tiab] OR "brain training"[tiab] OR "computer-based cognitive training"[tiab] OR "virtual reality cognitive training"[tiab] OR "multitasking training"[tiab] OR "dual task training"[tiab] OR "cognitive flexibility training"[tiab] OR "inhibitory control training"[tiab] OR "switching training"[tiab] OR "n-back training"[tiab] OR "updating training"[tiab] OR "shifting training"[tiab] OR "flexibility training"[tiab] OR "multi-domain training"[tiab] OR "cognitive adaptation training"[tiab] OR "brain fitness"[tiab] OR "brain games"[tiab] OR "computerized cognitive training"[tiab]) AND ("fMRI"[tiab] OR "functional magnetic resonance imaging"[tiab] OR "PET"[tiab] OR "positron emission tomography"[tiab] OR "SPECT"[tiab] OR "single photon emission computed tomography"[tiab] OR "functional imaging"[tiab] OR "neuroimaging"[tiab] OR "neuroplasticity"[tiab] OR "neural activation"[tiab] OR "brain function"[tiab])	700
MEDLINE	("cognitive training".ab,ti OR "cognitive rehabilitation".ab,ti OR "cognitive therapy".ab,ti OR "neuropsychological therapy".ab,ti OR "cognitive intervention".ab,ti OR "executive function training".ab,ti OR "working memory training".ab,ti OR "memory training".ab,ti OR "attention training".ab,ti OR "processing speed training".ab,ti OR "brain training".ab,ti OR "computer-based cognitive training".ab,ti OR "virtual reality cognitive training".ab,ti OR "multitasking training".ab,ti OR "dual task training".ab,ti OR "cognitive flexibility training".ab,ti OR "inhibitory control training".ab,ti OR "switching training".ab,ti OR "n-back training".ab,ti OR "updating training".ab,ti OR "shifting training".ab,ti OR "flexibility training".ab,ti OR "multi-domain training".ab,ti OR "cognitive adaptation training".ab,ti OR "brain fitness".ab,ti OR "brain games".ab,ti OR "computerized cognitive training".ab,ti) AND ("fMRI".ab,ti OR "functional magnetic resonance imaging".ab,ti OR "PET".ab,ti OR "positron emission tomography".ab,ti OR "SPECT".ab,ti OR "single photon emission computed tomography".ab,ti OR "functional imaging".ab,ti OR "neuroimaging".ab,ti OR "neuroplasticity".ab,ti OR "neural activation".ab,ti OR "brain function".ab,ti)	165
PsycINFO	("cognitive training".ti,ab OR "cognitive rehabilitation".ti,ab OR "cognitive therapy".ti,ab OR "neuropsychological therapy".ti,ab OR "cognitive intervention".ti,ab OR "executive function training".ti,ab OR "working memory training".ti,ab OR "memory training".ti,ab OR "attention training".ti,ab OR "processing speed training".ti,ab OR "brain training".ti,ab OR "computer-based cognitive training".ti,ab OR "virtual reality cognitive training".ti,ab OR "multitasking training".ti,ab OR "dual task training".ti,ab OR "cognitive flexibility training".ti,ab OR "inhibitory control training".ti,ab OR "switching training".ti,ab OR "n-back training".ti,ab OR "updating training".ti,ab OR "shifting training".ti,ab OR "flexibility training".ti,ab OR "multi-domain training".ti,ab OR "cognitive adaptation training".ti,ab OR "brain fitness".ti,ab OR "brain games".ti,ab OR "computerized cognitive training".ti,ab) AND ("fMRI".ti,ab OR "functional magnetic resonance imaging".ti,ab OR "PET".ti,ab OR "positron emission tomography".ti,ab OR "SPECT".ti,ab OR "single photon emission computed tomography".ti,ab OR "functional imaging".ti,ab OR "neuroimaging".ti,ab OR "neuroplasticity".ti,ab OR "neural activation".ti,ab OR "brain function".ti,ab)	1025
Embase	("cognitive training".ti,ab OR "cognitive rehabilitation".ti,ab OR "cognitive therapy".ti,ab OR "neuropsychological therapy".ti,ab OR "cognitive intervention".ti,ab OR "executive function training".ti,ab OR "working memory training".ti,ab OR "memory training".ti,ab OR "attention training".ti,ab OR "processing speed training".ti,ab OR "brain training".ti,ab OR "computer-based cognitive training".ti,ab OR "virtual reality cognitive training".ti,ab OR "multitasking training".ti,ab OR "dual task training".ti,ab OR "cognitive flexibility training".ti,ab OR "inhibitory control training".ti,ab OR "switching training".ti,ab OR "n-back training".ti,ab OR "updating training".ti,ab OR "shifting training".ti,ab OR "flexibility training".ti,ab OR "multi-domain training".ti,ab OR "cognitive adaptation training".ti,ab OR "brain fitness".ti,ab OR "brain games".ti,ab OR "computerized cognitive training".ti,ab) AND ("fMRI".ti,ab OR "functional magnetic resonance imaging".ti,ab OR "PET".ti,ab OR "positron emission	1334

Database	Search strategy	Records
	tomography".ti,ab OR "SPECT".ti,ab OR "single photon emission computed tomography".ti,ab OR "functional imaging".ti,ab OR "neuroimaging".ti,ab OR "neuroplasticity".ti,ab OR "neural activation".ti,ab OR "brain function".ti,ab)	
Web of Science	("cognitive training" OR "cognitive rehabilitation" OR "cognitive therapy" OR "neuropsychological therapy" OR "cognitive intervention" OR "executive function training" OR "working memory training" OR "memory training" OR "attention training" OR "processing speed training" OR "brain training" OR "computer-based cognitive training" OR "virtual reality cognitive training" OR "multitasking training" OR "dual task training" OR "cognitive flexibility training" OR "inhibitory control training" OR "switching training" OR "n-back training" OR "updating training" OR "shifting training" OR "flexibility training" OR "multi-domain training" OR "cognitive adaptation training" OR "brain fitness" OR "brain games" OR "computerized cognitive training") AND ("fMRI" OR "functional magnetic resonance imaging" OR "PET" OR "positron emission tomography" OR "SPECT" OR "single photon emission computed tomography" OR "functional imaging" OR "neuroimaging" OR "neuroplasticity" OR "neural activation" OR "brain function")	1435
Total		4659

Note. Database searches were conducted from inception to November 12, 2024. Reference lists of included studies and relevant reviews were additionally screened through June 20, 2026. Records are database-specific counts before final eligibility screening; search syntax was adapted to each database interface.

Supplementary Table S3. Characteristics of included studies.

No.	Study	Population (mean age)	Intervention group (n)	Intervention details	Control group (n)	In-scan tasks	Reporting-quality score
1	Alwashmi et al., 2024 [62]	Young adults (25.4)	Multi-sensory attention training (20)	Session duration: 30 minutes<br>Program length: 4 weeks<br>Training frequency: 5 times per week<br>Training domain: Multi-domain<br>Training paradigm: Process-based<br>Delivery mode: Individual-based<br>Difficulty regulation: NR	None	T1: Audio-visual voluntary task<br>T2: Visual-only voluntary task<br>T3: Audio-visual involuntary task	15.5
2	Ando et al., 2009 [63]	Young adults (22)	Working memory training (4)	Session duration: 12.5 minutes<br>Program length: 2 weeks<br>Training frequency: 7 times per week<br>Training domain: Single-domain<br>Training paradigm: Process-based<br>Delivery mode: Individual-based<br>Difficulty regulation: Non-adaptive	No training (4)	T1: Visuospatial working memory task with 30 visual targets<br>T2: Visuospatial working memory task with 10 visual targets	13.5
3	Beatty et al., 2015 [64]	Young adults (30.76)	Working memory training (22)	Session duration: 20 minutes<br>Program length: 1 week<br>Training frequency: 3 times per week<br>Training domain: Single-domain<br>Training paradigm: Process-based<br>Delivery mode: Individual-based<br>Difficulty regulation: Non-adaptive	Low-difficulty training (21)	Delayed matching-to-sample task	17.0
4	Berkman et al., 2014 [65]	Young adults (21.63)	Inhibitory control training (30)	Session duration: 6 minutes<br>Program length: 3 weeks<br>Training frequency: 3 times per week<br>Training domain: Single-domain<br>Training paradigm: Process-based<br>Delivery mode: Individual-based<br>Difficulty regulation: Adaptive	Low-difficulty training (30)	Stop signal task	16.0
5	Buschkuehl et al., 2014 [66]	Young adults (21.8)	Working memory training (27)	Session duration: 20 minutes<br>Program length: 1 week<br>Training frequency: 7 times per week<br>Training domain: Single-domain<br>Training paradigm: Process-based	Education program (28)	N-back task	16.0

No.	Study	Population (mean age)	Intervention group (n)	Intervention details	Control group (n)	In-scan tasks	Reporting-quality score
				Delivery mode: Individual-based<br>Difficulty regulation: Adaptive			
6	Chavan et al., 2015 [67]	Young adults (25.1)	Inhibit control training (18)	Session duration: 60 minutes<br>Program length: 2 weeks<br>Training frequency: 7 times per week<br>Training domain: Single-domain<br>Training paradigm: Process-based<br>Delivery mode: Individual-based<br>Difficulty regulation: Non-adaptive	None	T1: Go/nogo task<br>T2: N-back task	16.0
7	Clark et al., 2017 [68]	Young adults (30.68-31.33)	G1: Working memory training (25)<br>G2: Processing speed training (24-25)	Session duration: 20 minutes<br>Program length: 6 weeks<br>Training frequency: 5 times per week<br>Training domain: Single-domain<br>Training paradigm: Process-based<br>Delivery mode: Individual-based<br>Difficulty regulation: Adaptive for G1 Non-adaptive for G2	None	T1: Dual n-back task<br>T2: Raven's standard progressive matrices task<br>T3: Lexical decision task	15.5
8	Gaab et al., 2006 [69]	Young adults (26.5)	Verbal working memory training (7)	Session duration: 60 minutes<br>Program length: 1 week<br>Training frequency: 5 times per week<br>Training domain: Single-domain<br>Training paradigm: Process-based<br>Delivery mode: Individual-based<br>Difficulty regulation: Non-adaptive	None	Pitch memory task	16.5
9	Hiroyasu et al., 2015 [70]	Young adults (22.3)	G1: Working memory training with imagery strategy (4)<br>G2: Working memory training with rehearsal strategy (4-5)	Session duration: 10 minutes<br>Program length: 4 weeks<br>Training frequency: 5 times per week<br>Training domain: Single-domain<br>Training paradigm: Strategy-based<br>Delivery mode: Individual-based<br>Difficulty regulation: Non-adaptive	None	Reading span task	16.0
10	Jolles et al., 2010 [71]	Young adults (22.14)	Working memory training (16)	Session duration: 30 minutes<br>Program length: 2.5 weeks<br>Training frequency: 4.5 times per week	No training (16)	Verbal serial recall task	16.0

No.	Study	Population (mean age)	Intervention group (n)	Intervention details	Control group (n)	In-scan tasks	Reporting-quality score
				Training domain: Single-domain<br>Training paradigm: Process-based<br>Delivery mode: Individual-based<br>Difficulty regulation: Non-adaptive			
11	Kang et al., 2017 [72]	Young adults (22.1)	Cognitive control training (16)	Session duration: 30 minutes<br>Program length: 2 weeks<br>Training frequency: 4 times per week<br>Training domain: Multi-domain<br>Training paradigm: Strategy-based<br>Delivery mode: Individual-based<br>Difficulty regulation: Not Adaptive	None	Cued picture naming with language switching task	16.0
12	Kirschen et al., 2005 [73]	Young adults (25)	Working memory training (16)	Session duration: 12 minutes<br>Program length: 1 week<br>Training frequency: 1 times per week<br>Training domain: Single-domain<br>Training paradigm: Process-based<br>Delivery mode: Individual-based<br>Difficulty regulation: Non-adaptive	None	Sternberg task	15.5
13	Koch et al., 2006 [74]	Young adults (25.1)	Working memory training (10)	Session duration: 12 minutes<br>Program length: 1 week<br>Training frequency: 1 times per week<br>Training domain: Single-domain<br>Training paradigm: Process-based<br>Delivery mode: Individual-based<br>Difficulty regulation: Non-adaptive	None	Modified sternberg maintenance-retrieval task	15.5
14	Kondo et al., 2005 [75]	Young adults (22.3)	Episodic memory training (14)	Session duration: NR minutes<br>Program length: 1 week<br>Training frequency: 1 times per week<br>Training domain: Single-domain<br>Training paradigm: Strategy-based<br>Delivery mode: Individual-based<br>Difficulty regulation: Non-adaptive	None	Object memory task	15.5
15	Kühn et al., 2013 [76]	Young adults (25)	Working memory training (26)	Session duration: 166 minutes<br>Program length: NR weeks	Low-difficulty training (20)	Numerical memory updating task	17.0

No.	Study	Population (mean age)	Intervention group (n)	Intervention details	Control group (n)	In-scan tasks	Reporting-quality score
				Training frequency: 7 times per week<br>Training domain: Single-domain<br>Training paradigm: Process-based<br>Delivery mode: Individual-based<br>Difficulty regulation: Adaptive			
16	Landau et al., 2004 [77]	Young adults (24.5)	Working memory training (10)	Session duration: 30 minutes<br>Program length: 1 week<br>Training frequency: 7 times per week<br>Training domain: Single-domain<br>Training paradigm: Process-based<br>Delivery mode: Individual-based<br>Difficulty regulation: Non-adaptive	None	Face working memory task	16.0
17	Miró-Padilla et al., 2019 [78]	Young adults (22.6)	Working memory training (25)	Session duration: 50 minutes<br>Program length: 1 week<br>Training frequency: 4 times per week<br>Training domain: Single-domain<br>Training paradigm: Process-based<br>Delivery mode: Individual-based<br>Difficulty regulation: Adaptive	No training (27)	N-back task	17.0
18	Miró-Padilla et al., 2020 [79]	Young adults (22.6)	Working memory training (25)	Session duration: 45 minutes<br>Program length: 4 weeks<br>Training frequency: 3 times per week<br>Training domain: Single-domain<br>Training paradigm: Process-based<br>Delivery mode: Individual-based<br>Difficulty regulation: Adaptive	No training (27)	Paced auditory serial addition task	17.0
19	Olesen et al., 2004 [80]	Young adults (21.5-29.3)	Memory training (3-8)	Session duration: 40 minutes<br>Program length: 5 weeks<br>Training frequency: 7 times per week<br>Training domain: Single-domain<br>Training paradigm: Process-based<br>Delivery mode: Individual-based<br>Difficulty regulation: Adaptive	None	Working memory task	16.5

No.	Study	Population (mean age)	Intervention group (n)	Intervention details	Control group (n)	In-scan tasks	Reporting-quality score
20	Opitz et al., 2014 [81]	Young adults (23.67)	Verbal-to-visual code switching training (16)	Session duration: 30 minutes<br>Program length: 2 weeks<br>Training frequency: 4.5 times per week<br>Training domain: Single-domain<br>Training paradigm: Strategy-based<br>Delivery mode: Individual-based<br>Difficulty regulation: Adaptive	None	Reading span task	16.5
21	Schmicker et al., 2016 [82]	Young adults (24.3-24.4)	G1: Attention training (15)<br>G2: Memory training (14-15)	Session duration: 60 minutes<br>Program length: 1 week<br>Training frequency: 5 times per week<br>Training domain: Single-domain<br>Training paradigm: Process-based<br>Delivery mode: Individual-based<br>Difficulty regulation: Adaptive	None	Change detection task	14.5
22	Schneiders et al., 2012 [83]	Young adults (21.31)	Auditory working memory training (16)	Session duration: 50 minutes<br>Program length: 2 weeks<br>Training frequency: 4 times per week<br>Training domain: Single-domain<br>Training paradigm: Process-based<br>Delivery mode: Individual-based<br>Difficulty regulation: Adaptive	No training (16)	T1: Auditory 2-back task<br>T2: Visual 2-back task	17.0
23	Schweizer et al., 2013 [84]	Young adults (23)	Emotional working memory training (17)	Session duration: 25 minutes<br>Program length: NR weeks<br>Training frequency: 7 times per week<br>Training domain: Single-domain<br>Training paradigm: Process-based<br>Delivery mode: Individual-based<br>Difficulty regulation: Adaptive	Low-difficulty training (15)	Emotional 3-back task	17.0
24	Takeuchi et al., 2011 [85]	Young adults (21.62)	Attention training (23)	Session duration: 240 minutes<br>Program length: 1 week<br>Training frequency: 5 times per week<br>Training domain: Multi-domain<br>Training paradigm: Process-based	Low-difficulty training (21)	Language switching task	17.0

No.	Study	Population (mean age)	Intervention group (n)	Intervention details	Control group (n)	In-scan tasks	Reporting-quality score
				Delivery mode: Individual-based<br>Difficulty regulation: Non-adaptive			
25	Wagner et al., 2021 [86]	Young adults (24)	Working memory training (16-17)	Session duration: 30 minutes<br>Program length: 6 weeks<br>Training frequency: 7 times per week<br>Training domain: Single-domain<br>Training paradigm: Strategy-based<br>Delivery mode: Individual-based<br>Difficulty regulation: Adaptive	None	N-back task	15.0
26	Westerberg et al., 2007 [87]	Young adults (23)	G1:Memory training (17)<br>G2: Working memory training (3)	Session duration: 40 minutes<br>Program length: 5 weeks<br>Training frequency: 5 times per week<br>Training domain: Single-domain<br>Training paradigm: Strategy-based for G1<br>Process-based for G2<br>Delivery mode: Individual<br>Difficulty regulation: Adaptive	None	Word encoding / order judgment task	16.0
27	Zimmer et al., 2012 [88]	Young adults (31.5)	Working memory training (12)	Session duration: 40 minutes<br>Program length: 4 weeks<br>Training frequency: 2.5 times per week<br>Training domain: Single-domain<br>Training paradigm: Process-based<br>Delivery mode: Individual-based<br>Difficulty regulation: Adaptive	None	Change detection task	16.5
28	Dahlin et al., 2008 [89]	Young adults (23.59)	Updating training (15)	Session duration: 45 minutes<br>Program length: 5 weeks<br>Training frequency: 5 times per week<br>Training domain: Single-domain<br>Training paradigm: Process-based<br>Delivery mode: Individual-based<br>Difficulty regulation: Adaptive	No training (7)	T1: Letter memory task<br>T2: N-back task	16.5
29	Bless et al., 2014 [90]	Young adults (23.58)	Attention training (13)	Session duration: 12 minutes<br>Program length: 3 weeks<br>Training frequency: 7 times per week	No training (15)	Dichotic listening task	16.5

No.	Study	Population (mean age)	Intervention group (n)	Intervention details	Control group (n)	In-scan tasks	Reporting-quality score
				Training domain: Single-domain<br>Training paradigm: Process-based<br>Delivery mode: Individual-based<br>Difficulty regulation: Non-adaptive			
30	Lorenz et al., 2015 [91]	Young adults (23.6)	Multi-domain cognitive training (25)	Session duration: 30 minutes<br>Program length: 8 weeks<br>Training frequency: 7 times per week<br>Training domain: Multi-domain<br>Training paradigm: Process-based<br>Delivery mode: Individual-based<br>Difficulty regulation: Non-adaptive	No training (25)	Reward anticipation task	17.0
31	Lee et al., 2012 [92]	Young adults (21.16-21.68)	G1: Multi-domain training with hybrid variable-priority strategy (25)<br>G2: Multi-domain training with full emphasis strategy (25)	Session duration: 120 minutes<br>Program length: 4 weeks<br>Training frequency: 4 times per week<br>Training domain: Multi-domain<br>Training paradigm: Strategy-based<br>Delivery mode: Individual-based<br>Difficulty regulation: Non-adaptive	Low-difficulty training (25)	Ship-control task	16.0
32	Sacco et al., 2022 [93]	Young adults (24.21)	Memory training (23)	Session duration: 60 minutes<br>Program length: 1 week<br>Training frequency: 5 times per week<br>Training domain: Single-domain<br>Training paradigm: Process-based<br>Delivery mode: Individual-based<br>Difficulty regulation: Non-adaptive	None	Verbal memory task	16.5
33	Zhao et al., 2021 [94]	Young adults (21.89)	Working memory training (93)	Session duration: 35 minutes<br>Program length: 4 weeks<br>Training frequency: 5 times per week<br>Training domain: Single-domain<br>Training paradigm: Process-based<br>Delivery mode: Individual-based<br>Difficulty regulation: Adaptive	None	Visual-spatial span task	17.0
34	Hummer et al., 2019 [95]	Young adults (22.91-23.18)	G1: One-week game-based training (7)	Session duration: 75 minutes<br>Program length: 1 week	No training (3)	Go/nogo task	16.5

No.	Study	Population (mean age)	Intervention group (n)	Intervention details	Control group (n)	In-scan tasks	Reporting-quality score
			G2: Two-week game-based training (6-7)	Training frequency: 7 times per week<br>Training domain: Single-domain<br>Training paradigm: Process-based<br>Delivery mode: Individual-based<br>Difficulty regulation: Non-adaptive			
35	Beauchamp et al., 2016 [96]	Young adults (21.63)	Inhibitory control training (30)	Session duration: 6 minutes<br>Program length: 3 weeks<br>Training frequency: 3 times per week<br>Training domain: Single-domain<br>Training paradigm: Process-based<br>Delivery mode: Individual-based<br>Difficulty regulation: Adaptive	Sham training (30)	Cognitive reappraisal task	15.5
36	Flegal et al., 2019 [97]	Young adults (20.8)	Working memory updating training (19)	Session duration: 25 minutes<br>Program length: 3 weeks<br>Training frequency: 4 times per week<br>Training domain: Single-domain<br>Training paradigm: Process-based<br>Delivery mode: Individual-based<br>Difficulty regulation: Adaptive	No training (19)	T1: Matrix updating task<br>T2: Object-location association task<br>T3: N-back task	16.0
37	Chen et al., 2020 [98]	Young adults (22.88)	Language switching training (33)	Session duration: 30 minutes<br>Program length: 1 week<br>Training frequency: 8 times per week<br>Training domain: Single-domain<br>Training paradigm: Process-based<br>Delivery mode: Individual-based<br>Difficulty regulation: Adaptive	None	Picture naming task	17.0
38	Beauchamp et al., 2019 [99]	Adolescents (16.20-16.62)	Inhibitory control training (11)	Session duration: 6.5 minutes<br>Program length: 4.5 weeks<br>Training frequency: 2.5 times per week<br>Training domain: Single-domain<br>Training paradigm: Process-based<br>Delivery mode: Individual-based<br>Difficulty regulation: Non-adaptive	Active control condition (11)	T1: Stop-signal task<br>T2: Go/no-go task	16.0

No.	Study	Population (mean age)	Intervention group (n)	Intervention details	Control group (n)	In-scan tasks	Reporting-quality score
39	Conklin et al., 2015 [100]	Adolescents (11.90)	Working memory training (30)	Session duration: 37.5 minutes<br>Program length: 7 weeks<br>Training frequency: 5 times per week<br>Training domain: Single-domain<br>Training paradigm: Process-based<br>Delivery mode: Individual-based<br>Difficulty regulation: Adaptive	Usual care (30)	Working memory task	16.5
40	Cubillo et al., 2023 [101]	Primary school children (7.75)	Working memory training (16)	Session duration: 30 minutes<br>Program length: 5 weeks<br>Training frequency: 5 times per week<br>Training domain: Single-domain<br>Training paradigm: Process-based<br>Delivery mode: Individual-based<br>Difficulty regulation: Adaptive	Low-difficulty training (8)	T1: N-back task<br>T2: Flanker task	17.0
41	Kelly et al., 2020 [102]	Children (7.80)	Working memory training (23)	Session duration: 45 minutes<br>Program length: 6 weeks<br>Training frequency: 5 times per week<br>Training domain: Single-domain<br>Training paradigm: Process-based<br>Delivery mode: Individual-based<br>Difficulty regulation: Adaptive	None	Working memory task	16.0
42	Kesler et al., 2011 [103]	Children (10.90)	Cognitive rehabilitation / executive-function training (16)	Session duration: 20 minutes<br>Program length: 6 weeks<br>Training frequency: 5 times per week<br>Training domain: Single-domain<br>Training paradigm: Process-based<br>Delivery mode: Individual-based<br>Difficulty regulation: Adaptive	None	Executive-function task	16.0
43	Rosenberg-Lee et al., 2018 [104]	Children (8.63)	Strategy-based cognitive training (19)	Session duration: 45 minutes<br>Program length: 8 weeks<br>Training frequency: 3 times per week<br>Training domain: Single-domain<br>Training paradigm: Strategy-based	Reading (15)	Cognitive control task	16.0

No.	Study	Population (mean age)	Intervention group (n)	Intervention details	Control group (n)	In-scan tasks	Reporting-quality score
				Delivery mode: Individual-based<br>Difficulty regulation: Non-adaptive			
44	Liu et al., 2018 [105]	Children (10.79)	Executive-function training (43)	Session duration: 15 minutes<br>Program length: 4 weeks<br>Training frequency: 1 times per week<br>Training domain: Single-domain<br>Training paradigm: Process-based<br>Delivery mode: Individual-based<br>Difficulty regulation: Non-adaptive	Low-difficulty training (41)	Cognitive control task	16.0
45	Miotto et al., 2020 [106]	Children/adolescents (9.50; 15.80)	Verbal episodic memory strategy training (12-13)	Session duration: 30 minutes<br>Program length: 8 weeks<br>Training frequency: 1 times per week<br>Training domain: Single-domain<br>Training paradigm: Strategy-based<br>Delivery mode: Group-based<br>Difficulty regulation: Non-adaptive	None	Word-list encoding and recognition task	17.0
46	Newman et al., 2016 [107]	Children (8.17-8.25)	Block play / board game cognitive training (14)	Session duration: 30 minutes<br>Program length: 5 weeks<br>Training frequency: 1 time per week<br>Training domain: Single-domain<br>Training paradigm: Process-based<br>Delivery mode: Group-based<br>Difficulty regulation: Non-adaptive	None	Spatial cognition task	14.0
47	Stevens et al., 2016 [108]	Adolescents (15.20)	Working memory training (18)	Session duration: 30 minutes<br>Program length: 5 weeks<br>Training frequency: 5 times per week<br>Training domain: Single-domain<br>Training paradigm: Process-based<br>Delivery mode: Individual-based<br>Difficulty regulation: Adaptive	None	Working memory task	16.0
48	Wang et al., 2019 [109]	Children (11.94)	Abacus-based mental calculation (62)	Session duration: 60 minutes<br>Program length: 180 weeks<br>Training frequency: 2 times per week<br>Training domain: Single-domain	Reading (56)	N-back task	17.0

No.	Study	Population (mean age)	Intervention group (n)	Intervention details	Control group (n)	In-scan tasks	Reporting-quality score
				Training paradigm: Strategy-based<br>Delivery mode: Individual-based<br>Difficulty regulation: Adaptive			
49	Jolles et al., 2012 [110]	Young adults (22.04)	Working memory training (15)	Session duration: 25 minutes<br>Program length: 6 weeks<br>Training frequency: 2.5 times per week<br>Training domain: Single-domain<br>Training paradigm: Process-based<br>Delivery mode: Individual-based<br>Difficulty regulation: Adaptive	None	N-back task	17.0
50	Jolles et al., 2012 [110]	Children (12.35)	Working memory training (10)	Session duration: 25 minutes<br>Program length: 6 weeks<br>Training frequency: 2.5 times per week<br>Training domain: Single-domain<br>Training paradigm: Process-based<br>Delivery mode: Individual-based<br>Difficulty regulation: Adaptive	None	Emotional n-back task	17.0
51	Kable et al., 2017 [111]	Young adults (25.1)	Multi-domain training (64)	Session duration: 30 minutes<br>Program length: 10 weeks<br>Training frequency: 5 times per week<br>Training domain: Multi-domain<br>Training paradigm: Process-based<br>Delivery mode: Individual-based<br>Difficulty regulation: Adaptive	Mental leisure activities (64)	Delay discounting task	17.0
52	Nęcka et al., 2021 [112]	Young adults (27.2)	Working memory training (23)	Session duration: 32.5 minutes<br>Program length: 2 weeks<br>Training frequency: 5 times per week<br>Training domain: Single-domain<br>Training paradigm: Process-based<br>Delivery mode: Individual-based<br>Difficulty regulation: Adaptive	No training (23)	T1: N-back task<br>T2: Stop-signal task	16.0
53	Wu et al., 2021 [113]	Young adults (22.3)	Attention training (20)	Session duration: 30 minutes<br>Program length: 1 week<br>Training frequency: 8 times per week	Low-difficulty training (39)	Language switching task	17.0

No.	Study	Population (mean age)	Intervention group (n)	Intervention details	Control group (n)	In-scan tasks	Reporting-quality score
				Training domain: Single-domain<br>Training paradigm: Process-based<br>Delivery mode: Individual-based<br>Difficulty regulation: Non-adaptive			
54	Ganesan et al., 2024 [114]	Children (8.97)	Cognitive training (70)	Session duration: 15 minutes<br>Program length: 8 weeks<br>Training frequency: 4 times per week<br>Training domain: Single-domain<br>Training paradigm: Process-based<br>Delivery mode: Individual-based<br>Difficulty regulation: Adaptive	Low-difficulty training (66)	Task-activation fMRI contrast with no reported significant coordinates	16.5
55	Jones et al., 2022 [115]	Children/adolescents (12.17)	Working memory training (17)	Session duration: 45 minutes<br>Program length: 5 weeks<br>Training frequency: 5 times per week<br>Training domain: Single-domain<br>Training paradigm: Process-based<br>Delivery mode: Individual-based<br>Difficulty regulation: Adaptive	Low-difficulty training (15)	Task-activation fMRI contrast with no reported significant coordinates	16.5
56	Ando et al., 2007 [116]	Young adults (21.5)	Working memory training (2)	Session duration: 12.5 minutes<br>Program length: 1 week<br>Training frequency: 7 times per week<br>Training domain: Single-domain<br>Training paradigm: Process-based<br>Delivery mode: Individual-based<br>Difficulty regulation: Non-adaptive	No training (3)	T1: Visuospatial working memory task with 30 visual targets<br>T2: Visuospatial working memory task with 10 visual targets	13.5
57	Garavan et al., 2000 [117]	Young adults (26)	Working memory training (12)	Session duration: 20 minutes<br>Program length: 1 week<br>Training frequency: 1 times per week<br>Training domain: Single-domain<br>Training paradigm: Process-based<br>Delivery mode: Individual-based<br>Difficulty regulation: Non-adaptive	None	Delayed match-to-sample task	15.5
58	Salminen et al., 2016 [118]	Young adults (24.1-24.4)	G1: Dual-task working memory training	Session duration: 30 minutes<br>Program length: 2.29 weeks	None	T1: Dual n-back task<br>T2: Working memory updating task	17.0

No.	Study	Population (mean age)	Intervention group (n)	Intervention details	Control group (n)	In-scan tasks	Reporting-quality score
			(18)<br>G2: Working memory training (18)	Training frequency: 7 times per week<br>Training domain: Single-domain<br>Training paradigm: Process-based<br>Delivery mode: Individual-based<br>Difficulty regulation: Adaptive			
59	Vartanian et al., 2013 [119]	Young adults (30.79)	Working memory training (17)	Session duration: 20 minutes<br>Program length: 1 week<br>Training frequency: 3 times per week<br>Training domain: Single-domain<br>Training paradigm: Process-based<br>Delivery mode: Individual-based<br>Difficulty regulation: Non-adaptive	Low-difficulty training (17)	Alternate uses task	15.5
60	Hoekzema et al., 2010 [120]	Children (11.10)	Attention / executive-function training (9)	Session duration: 45 minutes<br>Program length: 2 weeks<br>Training frequency: 5 times per week<br>Training domain: Single-domain<br>Training paradigm: Process-based<br>Delivery mode: Group-based<br>Difficulty regulation: Non-adaptive	None	T1: Response inhibition task<br>T2: Selective attention task	13.5
61	Hong et al., 2015 [121]	Children/adolescents (NR)	Working memory training (8)	Session duration: 30 minutes<br>Program length: 8 weeks<br>Training frequency: 2.5 times per week<br>Training domain: Single-domain<br>Training paradigm: Process-based<br>Delivery mode: Individual-based<br>Difficulty regulation: Adaptive	None	Working memory task	15.0
62	Iuculano et al., 2015 [122]	Children (8.65)	Math learning / cognitive tutoring (15)	Session duration: NR minutes<br>Program length: 8 weeks<br>Training frequency: NR times per week<br>Training domain: Single-domain<br>Training paradigm: Process-based<br>Delivery mode: Individual-based<br>Difficulty regulation: Non-adaptive	None	Arithmetic / numerical cognition task	17.0

No.	Study	Population (mean age)	Intervention group (n)	Intervention details	Control group (n)	In-scan tasks	Reporting-quality score
63	Kucian et al., 2011 [123]	Children (9.50)	Numerical cognition training (32)	Session duration: 14.8 minutes<br>Program length: 5 weeks<br>Training frequency: 4.85 times per week<br>Training domain: Single-domain<br>Training paradigm: Process-based<br>Delivery mode: Individual-based<br>Difficulty regulation: Adaptive	None	Arithmetic / numerical cognition task	14.5
64	Murdaugh et al., 2016 [124]	Children/adolescents (10.95)	Cognitive / imagery-based intervention (13)	Session duration: 240 minutes<br>Program length: 10 weeks<br>Training frequency: 5 times per week<br>Training domain: Single-domain<br>Training paradigm: Process-based<br>Delivery mode: Individual-based<br>Difficulty regulation: Non-adaptive	Waitlist (13)	High-imagery task	16.0
65	de Oliveira Rosa et al., 2020 [125]	Children/adolescents (11.40)	Adaptive cognitive training (10)	Session duration: 30 minutes<br>Program length: 12 weeks<br>Training frequency: 4 times per week<br>Training domain: Single-domain<br>Training paradigm: Process-based<br>Delivery mode: Individual-based<br>Difficulty regulation: Adaptive	Low-difficulty training (10)	T1: Sustained attention task<br>T2: Working memory task<br>T3: Go/no-go task	16.0

Supplementary Table S4. Reporting-quality assessment checklist.

**Subjects**
1. The sample size was appropriate
2. Subjects were evaluated prospectively, demographic data were reported (age (mean and SD/range), sex, and handedness), and psychiatric and medical illnesses were excluded
3. If any subject was scanned but then rejected from the analysis, withdrawals from the study were explained
Methods for tasks
4. All participants went through a training session outside the scanner
5. The experimental design was meticulously detailed to enable replication, including the number of blocks or trials per participant, the duration of each trial, the inter-stimulus interval, and whether the design was block-based or event-related.
6. The stimuli and the number of repetitions were sufficient and clearly described
7. If applicable, the baseline condition was defined as almost the same as the task condition.
Methods for image acquisition and statistical analysis
8. MRI slice thickness ≤ 3 mm
9. 3T MRI was used
10. The imaging technique used for data acquisition was clearly described so that it could be reproduced (e.g., MRI system used, field strength, pulse sequence type, number of volumes per session, field of view, matrix size, slice thickness, interslice skip, acquisition orientation, TE/TR/flip angle)
11. Preprocessing operations were clearly described and detailed so that they could be reproduced (e.g., software used, order of preprocessing operations, slice-timing, motion correction, coregistration and normalization (linear/affine or nonlinear), smoothing)
12. Adjustments were made for multiple statistical comparisons
13. Appropriate design and/or analytical methods to control confounding
14. Appropriate use of statistics for primary analysis effect (excluding control of confounders)
Results, conclusions, and conflicts of interest
15. Statistical parameters for significant and important nonsignificant differences were provided

Subjects
16. Conclusions were consistent with the results obtained and the limitations were discussed
17. Declarations of conflicts of interest or identification of funding sources

Note. The checklist was adapted from Lavallée et al. [61] to assess reporting completeness and technical methodological features; it is not a formal risk-of-bias instrument. Each item was scored 0, 0.5, or 1. Final study-level scores are provided in Supplementary Table S3.

Supplementary Table S5. Item-level reporting-quality ratings for the included studies.

Study	1	2	3	4	5	6	7	8	9	10	11	12	13	14	15	16	17
Alwashmi et al., 2024 [62]	1	0.5	0.5	1	1	1	1	1	1	1	1	1	0.5	1	1	1	1
Ando et al., 2009 [63]	1	0.5	0.5	1	0.5	0.5	0.5	1	1	1	1	0.5	0.5	1	1	1	1
Beatty et al., 2015 [64]	1	1	1	1	1	1	1	1	1	1	1	1	1	1	1	1	1
Berkman et al., 2014 [65]	1	0.5	0.5	1	1	1	1	1	1	1	1	1	1	1	1	1	1
Buschkuehl et al., 2014 [66]	1	0.5	0.5	1	1	1	1	1	1	1	1	1	1	1	1	1	1
Chavan et al., 2015 [67]	1	1	0.5	1	1	1	1	1	1	1	1	1	0.5	1	1	1	1
Clark et al., 2017 [68]	1	0.5	0.5	1	1	1	1	1	1	1	1	1	0.5	1	1	1	1
Gaab et al., 2006 [69]	1	1	1	1	1	1	1	1	1	1	1	1	0.5	1	1	1	1
Hiroyasu et al., 2015 [70]	1	1	0.5	1	1	1	1	1	1	1	1	1	0.5	1	1	1	1
Jolles et al., 2010 [71]	1	0.5	0.5	1	1	1	1	1	1	1	1	1	1	1	1	1	1
Kang et al., 2017 [72]	1	1	0.5	1	1	1	1	1	1	1	1	1	0.5	1	1	1	1
Kirschen et al., 2005 [73]	1	0.5	0.5	1	1	1	1	1	1	1	1	1	0.5	1	1	1	1
Koch et al., 2006 [74]	1	0.5	0.5	1	1	1	1	1	1	1	1	1	0.5	1	1	1	1
Kondo et al., 2005 [75]	1	1	1	1	0.5	0.5	1	1	1	1	1	1	0.5	1	1	1	1
Kühn et al., 2013 [76]	1	1	1	1	1	1	1	1	1	1	1	1	1	1	1	1	1
Landau et al., 2004 [77]	1	1	0.5	1	1	1	1	1	1	1	1	1	0.5	1	1	1	1
Miró-Padilla et al., 2019 [78]	1	1	1	1	1	1	1	1	1	1	1	1	1	1	1	1	1
Miró-Padilla et al., 2020 [79]	1	1	1	1	1	1	1	1	1	1	1	1	1	1	1	1	1
Olesen et al., 2004 [80]	1	1	1	1	1	1	1	1	1	1	1	1	0.5	1	1	1	1
Opitz et al., 2014 [81]	1	1	1	1	1	1	1	1	1	1	1	1	0.5	1	1	1	1
Schmicker et al., 2016 [82]	1	0.5	0.5	1	0.5	0.5	1	1	1	1	1	1	0.5	1	1	1	1
Schneiders et al., 2012 [83]	1	1	1	1	1	1	1	1	1	1	1	1	1	1	1	1	1
Schweizer et al., 2013 [84]	1	1	1	1	1	1	1	1	1	1	1	1	1	1	1	1	1
Takeuchi et al., 2011 [85]	1	1	1	1	1	1	1	1	1	1	1	1	1	1	1	1	1
Wagner et al., 2021 [86]	1	0.5	0.5	1	1	1	1	1	1	1	1	1	0.5	1	1	0.5	1
Westerberg et al., 2007 [87]	1	1	0.5	1	1	1	1	1	1	1	1	1	0.5	1	1	1	1
Zimmer et al., 2012 [88]	1	1	1	1	1	1	1	1	1	1	1	1	0.5	1	1	1	1
Dahlin et al., 2008 [89]	1	0.5	1	1	1	1	1	1	1	1	1	1	1	1	1	1	1
Bless et al., 2014 [90]	1	1	0.5	1	1	1	1	1	1	1	1	1	1	1	1	1	1
Lorenz et al., 2015 [91]	1	1	1	1	1	1	1	1	1	1	1	1	1	1	1	1	1
Lee et al., 2012 [92]	1	0.5	0.5	1	1	1	1	1	1	1	1	1	1	1	1	1	1
Sacco et al., 2022 [93]	1	1	1	1	1	1	1	1	1	1	1	1	0.5	1	1	1	1
Zhao et al., 2021 [94]	1	1	1	1	1	1	1	1	1	1	1	1	1	1	1	1	1
Hummer et al., 2019 [95]	1	0.5	1	1	1	1	1	1	1	1	1	1	1	1	1	1	1
Beauchamp et al., 2016 [96]	1	0.5	0.5	1	1	1	1	1	1	1	1	1	0.5	1	1	1	1
Flegal et al., 2019 [97]	1	0.5	0.5	1	1	1	1	1	1	1	1	1	1	1	1	1	1
Chen et al., 2020 [98]	1	1	1	1	1	1	1	1	1	1	1	1	1	1	1	1	1
Beauchamp et al., 2019 [99]	0.5	0.5	1	1	1	1	1	1	1	1	1	1	1	1	1	1	1
Conklin et al., 2015 [100]	1	0.5	1	1	1	1	1	1	1	1	1	1	1	1	1	1	1
Cubillo et al., 2023 [101]	1	1	1	1	1	1	1	1	1	1	1	1	1	1	1	1	1
Kelly et al., 2020 [102]	0.5	0.5	1	1	1	1	1	1	1	1	1	1	1	1	1	1	1
Kesler et al., 2011 [103]	0.5	0.5	1	1	1	1	1	1	1	1	1	1	1	1	1	1	1
Rosenberg-Lee et al., 2018 [104]	0.5	0.5	1	1	1	1	1	1	1	1	1	1	1	1	1	1	1
Liu et al., 2018 [105]	1	0.5	1	1	1	1	1	1	1	1	1	1	0.5	1	1	1	1
Miotto et al., 2020 [106]	1	1	1	1	1	1	1	1	1	1	1	1	1	1	1	1	1
Newman et al., 2016 [107]	0.5	0.5	0.5	1	0.5	1	1	1	1	1	1	0.5	0.5	1	1	1	1
Stevens et al., 2016 [108]	0.5	0.5	1	1	1	1	1	1	1	1	1	1	1	1	1	1	1
Wang et al., 2019 [109]	1	1	1	1	1	1	1	1	1	1	1	1	1	1	1	1	1
Jolles et al., 2012 [110]	1	1	1	1	1	1	1	1	1	1	1	1	1	1	1	1	1
Kable et al., 2017 [111]	1	1	1	1	1	1	1	1	1	1	1	1	1	1	1	1	1
Nęcka et al., 2021 [112]	1	0.5	0.5	1	1	1	1	1	1	1	1	1	1	1	1	1	1
Wu et al., 2021 [113]	1	1	1	1	1	1	1	1	1	1	1	1	1	1	1	1	1
Ganesan et al., 2024 [114]	1	0.5	1	1	1	1	1	1	1	1	1	1	1	1	1	1	1
Jones et al., 2022 [115]	1	0.5	1	1	1	1	1	1	1	1	1	1	1	1	1	1	1
Ando et al., 2007 [116]	1	0.5	0.5	1	0.5	0.5	0.5	1	1	1	1	0.5	0.5	1	1	1	1
Garavan et al., 2000 [117]	1	0.5	0.5	1	1	1	1	1	1	1	1	1	0.5	1	1	1	1
Salminen et al., 2016 [118]	1	1	1	1	1	1	1	1	1	1	1	1	1	1	1	1	1
Vartanian et al., 2013 [119]	1	0.5	0.5	1	1	1	1	1	1	1	1	1	0.5	1	1	1	1
Hoekzema et al., 2010 [120]	0.5	0.5	0.5	1	0.5	0.5	1	1	1	1	1	0.5	0.5	1	1	1	1
Hong et al., 2015 [121]	0.5	0.5	0.5	1	1	1	1	1	1	1	1	1	0.5	1	1	1	1
Iuculano et al., 2015 [122]	1	1	1	1	1	1	1	1	1	1	1	1	1	1	1	1	1
Kucian et al., 2011 [123]	0.5	0.5	0.5	1	1	1	1	0.5	1	1	1	1	0.5	1	1	1	1
Murdaugh et al., 2016 [124]	0.5	0.5	1	1	1	1	1	1	1	1	1	1	1	1	1	1	1
de Oliveira Rosa et al., 2020 [125]	0.5	0.5	1	1	1	1	1	1	1	1	1	1	1	1	1	1	1

Note. Q1–Q17 correspond to the checklist items in Supplementary Table S4. Ratings are 0, 0.5, or 1. Two authors (TF and CL) independently assessed all included studies at the item level using the 17-item reporting-quality checklist, with disagreements resolved through discussion.

Supplementary Table S6. Jackknife analysis for the overall main SDM analysis.

Study omitted	R SPL (18,−64,54)	R OP/V1 (14,−94,6)	L cerebellum lobule IV/V (−6,−50,0)	R posterior STG (60,−32,10)	L ACC/paracingulate (−2,28,28)	R insula (44,20,−2)	L PCC (−2,−34,30)
Conklin et al., 2015 [100]	Yes	Yes	Yes	Yes	Yes	Yes	Yes
Hoekzema et al., 2010 [120]	Yes	Yes	Yes	Yes	Yes	Yes	Yes
Jolles et al., 2012 [110]	Yes	Yes	Yes	Yes	Yes	Yes	Yes
Kelly et al., 2020 [102]	Yes	Yes	Yes	Yes	Yes	Yes	Yes
Newman et al., 2016 [107]	Yes	Yes	Yes	Yes	Yes	Yes	Yes
Stevens et al., 2016 [108]	Yes	Yes	Yes	Yes	Yes	Yes	Yes
Rosenberg-Lee et al., 2018 [104]	Yes	Yes	Yes	Yes	Yes	Yes	Yes
Wang et al., 2019 [109]	Yes	Yes	Yes	Yes	Yes	Yes	Yes
Liu et al., 2018 [105]	Yes	Yes	Yes	Yes	Yes	Yes	Yes
Beauchamp et al., 2019 [99]	Yes	Yes	Yes	Yes	Yes	Yes	Yes
Kesler et al., 2011 [103]	Yes	Yes	Yes	Yes	Yes	Yes	Yes
de Oliveira Rosa et al., 2020 [125]	Yes	Yes	Yes	Yes	Yes	Yes	Yes
Miotto et al., 2020 [106]	Yes	Yes	Yes	Yes	Yes	Yes	Yes
Cubillo et al., 2023 [101]	Yes	Yes	Yes	Yes	Yes	Yes	Yes
Hong et al., 2015 [121]	Yes	Yes	Yes	Yes	Yes	Yes	Yes
Iuculano et al., 2015 [122]	Yes	Yes	Yes	Yes	Yes	Yes	Yes
Murdaugh et al., 2016 [124]	Yes	Yes	Yes	Yes	Yes	Yes	Yes

Study omitted	R SPL (18,−64,54)	R OP/V1 (14,−94,6)	L cerebellum lobule IV/V (−6,−50,0)	R posterior STG (60,−32,10)	L ACC/paracingulate (−2,28,28)	R insula (44,20,−2)	L PCC (−2,−34,30)
Kucian et al., 2011 [123]	Yes	Yes	Yes	Yes	Yes	Yes	Yes
Jones et al., 2022 [115]	Yes	Yes	Yes	Yes	Yes	Yes	Yes
Ganesan et al., 2024 [114]	Yes	Yes	Yes	Yes	Yes	Yes	Yes
Alwashmi et al., 2024 [62]	Yes	Yes	Yes	Yes	Yes	Yes	Yes
Ando et al., 2007 [116]	Yes	Yes	Yes	Yes	Yes	Yes	Yes
Ando et al., 2009 [63]	Yes	Yes	Yes	Yes	Yes	Yes	Yes
Beatty et al., 2015 [64]	Yes	Yes	Yes	Yes	Yes	Yes	Yes
Berkman et al., 2014 [65]	Yes	Yes	Yes	Yes	Yes	Yes	Yes
Buschkuehl et al., 2014 [66]	Yes	Yes	Yes	Yes	Yes	Yes	Yes
Chavan et al., 2015 [67]	Yes	Yes	Yes	Yes	Yes	Yes	Yes
Clark et al., 2017 [68]	Yes	Yes	Yes	Yes	Yes	Yes	Yes
Gaab et al., 2006 [69]	Yes	Yes	Yes	Yes	Yes	Yes	Yes
Garavan et al., 2000 [117]	Yes	Yes	Yes	Yes	Yes	Yes	Yes
Hiroyasu et al., 2015 [70]	Yes	Yes	Yes	Yes	Yes	Yes	Yes
Jolles et al., 2010 [71]	Yes	Yes	Yes	Yes	Yes	Yes	Yes
Kable et al., 2017 [111]	Yes	Yes	Yes	Yes	Yes	Yes	Yes
Kang et al., 2017 [72]	Yes	Yes	Yes	Yes	Yes	Yes	Yes
Kirschen et al., 2005 [73]	Yes	Yes	Yes	Yes	Yes	Yes	Yes
Koch et al., 2006 [74]	Yes	Yes	Yes	Yes	Yes	Yes	Yes

Study omitted	R SPL (18,−64,54)	R OP/V1 (14,−94,6)	L cerebellum lobule IV/V (−6,−50,0)	R posterior STG (60,−32,10)	L ACC/paracingulate (−2,28,28)	R insula (44,20,−2)	L PCC (−2,−34,30)
Kondo et al., 2005 [75]	Yes	Yes	Yes	Yes	Yes	Yes	Yes
Kuhn et al., 2013 [76]	Yes	Yes	Yes	Yes	Yes	Yes	Yes
Landau et al., 2004 [77]	Yes	Yes	Yes	Yes	Yes	Yes	Yes
Miró-Padilla et al., 2019 [78]	Yes	Yes	Yes	Yes	Yes	Yes	Yes
Miró-Padilla et al., 2020 [79]	Yes	Yes	Yes	Yes	Yes	Yes	Yes
Olesen et al., 2004 [80]	Yes	Yes	Yes	Yes	Yes	Yes	Yes
Opitz et al., 2014 [81]	Yes	Yes	Yes	Yes	Yes	Yes	Yes
Salminen et al., 2016 [118]	Yes	Yes	Yes	Yes	Yes	Yes	Yes
Schmicker et al., 2016 [82]	Yes	Yes	Yes	Yes	Yes	Yes	Yes
Schneiders et al., 2012 [83]	Yes	Yes	Yes	Yes	Yes	Yes	Yes
Schweizer et al., 2013 [84]	Yes	Yes	Yes	Yes	Yes	Yes	Yes
Takeuchi et al., 2011 [85]	Yes	Yes	Yes	Yes	Yes	Yes	Yes
Vartanian et al., 2013 [119]	Yes	Yes	Yes	Yes	Yes	Yes	Yes
Wagner et al., 2021 [86]	Yes	Yes	Yes	Yes	Yes	Yes	Yes
Westerberg et al., 2007 [87]	Yes	Yes	Yes	Yes	Yes	Yes	Yes
Zimmer et al., 2012 [88]	Yes	Yes	Yes	Yes	Yes	Yes	Yes
Dahlin et al., 2008 [89]	Yes	Yes	Yes	Yes	Yes	Yes	Yes
Bless et al., 2014 [90]	Yes	Yes	Yes	Yes	Yes	Yes	Yes
Lorenz et al., 2015 [91]	Yes	Yes	Yes	Yes	Yes	Yes	Yes

Study omitted	R SPL (18,−64,54)	R OP/V1 (14,−94,6)	L cerebellum lobule IV/V (−6,−50,0)	R posterior STG (60,−32,10)	L ACC/paracingulate (−2,28,28)	R insula (44,20,−2)	L PCC (−2,−34,30)
Lee et al., 2012 [92]	Yes	Yes	Yes	Yes	Yes	Yes	Yes
Sacco et al., 2022 [93]	Yes	Yes	Yes	Yes	Yes	Yes	Yes
Zhao et al., 2021 [94]	Yes	Yes	Yes	Yes	Yes	Yes	Yes
Hummer et al., 2019 [95]	Yes	Yes	Yes	Yes	Yes	Yes	Yes
Beauchamp et al., 2016 [96]	Yes	Yes	Yes	Yes	Yes	Yes	Yes
Flegal et al., 2019 [97]	Yes	Yes	Yes	Yes	Yes	Yes	Yes
Chen et al., 2020 [98]	Yes	Yes	Yes	Yes	Yes	Yes	Yes
Wu et al., 2021 [113]	Yes	Yes	Yes	Yes	Yes	Yes	Yes
Nęcka et al., 2021 [112]	Yes	Yes	Yes	Yes	Yes	Yes	Yes

Note. Rows are unique study-level jackknife units, and columns are significant clusters from the overall main SDM analysis. Yes indicates that the cluster remained significant after the omitted study was removed. Multiple dependent task contrasts from the same study were removed together. Threshold: voxel-wise $p < 0.005$, absolute SDM-Z > 1, and cluster extent $k \geq 100$ voxels in MNI space. Bracketed citation numbers refer to the main-text reference list.

Supplementary Table S7. Jackknife analysis for the children/adolescents main SDM analysis.

Study omitted	R cuneus (16,−96,6)	R MFG (32,50,22)	L OP/V1 (−14,−92,0)	R IPL (44,−34,46)	L SMA (0,18,48)	L MFG (−34,28,30)	L insula (−42,16,−4)
Conklin et al., 2015 [100]	Yes	Yes	Yes	Yes	Yes	Yes	Yes
Hoekzema et al., 2010 [120]	Yes	Yes	Yes	Yes	Yes	Yes	Yes
Jolles et al., 2012 [110]	Yes	Yes	Yes	Yes	Yes	Yes	Yes
Kelly et al., 2020 [102]	Yes	Yes	Yes	Yes	Yes	Yes	Yes
Newman et al., 2016 [107]	Yes	Yes	Yes	Yes	Yes	Yes	Yes
Stevens et al., 2016 [108]	Yes	Yes	Yes	Yes	Yes	Yes	Yes
Rosenberg-Lee et al., 2018 [104]	Yes	Yes	Yes	Yes	Yes	Yes	Yes
Wang et al., 2019 [109]	Yes	Yes	Yes	Yes	Yes	Yes	Yes
Liu et al., 2018 [105]	Yes	Yes	Yes	Yes	Yes	Yes	Yes
Beauchamp et al., 2019 [99]	Yes	Yes	Yes	Yes	Yes	Yes	Yes
Kesler et al., 2011 [103]	Yes	Yes	Yes	Yes	Yes	Yes	Yes
de Oliveira Rosa et al., 2020 [125]	Yes	Yes	Yes	Yes	Yes	Yes	Yes
Miotto et al., 2020 [106]	Yes	Yes	Yes	Yes	Yes	Yes	Yes
Cubillo et al., 2023 [101]	Yes	Yes	Yes	Yes	Yes	Yes	Yes
Hong et al., 2015 [121]	Yes	Yes	Yes	Yes	Yes	Yes	Yes
Iuculano et al., 2015 [122]	Yes	Yes	Yes	Yes	Yes	Yes	Yes
Murdaugh et al., 2016 [124]	Yes	Yes	Yes	Yes	Yes	Yes	Yes
Kucian et al., 2011 [123]	Yes	Yes	Yes	Yes	Yes	Yes	Yes

Study omitted	R cuneus (16,−96,6)	R MFG (32,50,22)	L OP/V1 (−14,−92,0)	R IPL (44,−34,46)	L SMA (0,18,48)	L MFG (−34,28,30)	L insula (−42,16,−4)
Jones et al., 2022 [115]	Yes	Yes	Yes	Yes	Yes	Yes	Yes
Ganesan et al., 2024 [114]	Yes	Yes	Yes	Yes	Yes	Yes	Yes

Note. Rows are unique study-level jackknife units, and columns are significant clusters from the children/adolescents main SDM analysis. Yes indicates that the cluster remained significant after the omitted study was removed. Multiple dependent task contrasts from the same study were removed together. Threshold: voxel-wise $p < 0.005$, absolute SDM-Z > 1, and cluster extent $k \geq 100$ voxels in MNI space. Bracketed citation numbers refer to the main-text reference list.

Supplementary Table S8. Jackknife analysis for the young-adult main SDM analysis.

Study omitted	R SPL/precuneus (18,−56,50)	L ACC/paracingulate (−2,34,24)	R insula (46,22,−2)	L PCC (−4,−38,30)
Alwashmi et al., 2024 [62]	Yes	Yes	Yes	Yes
Ando et al., 2007 [116]	Yes	Yes	Yes	Yes
Ando et al., 2009 [63]	Yes	Yes	Yes	Yes
Beatty et al., 2015 [64]	Yes	Yes	Yes	Yes
Berkman et al., 2014 [65]	Yes	Yes	Yes	Yes
Buschkuehl et al., 2014 [66]	Yes	Yes	Yes	Yes
Chavan et al., 2015 [67]	Yes	Yes	Yes	Yes
Clark et al., 2017 [68]	Yes	Yes	Yes	Yes
Gaab et al., 2006 [69]	Yes	Yes	Yes	Yes
Garavan et al., 2000 [117]	Yes	Yes	Yes	Yes
Hiroyasu et al., 2015 [70]	Yes	Yes	Yes	Yes
Jolles et al., 2010 [71]	Yes	Yes	Yes	Yes
Jolles et al., 2012 [110]	Yes	Yes	Yes	Yes
Kable et al., 2017 [111]	Yes	Yes	Yes	Yes
Kang et al., 2017 [72]	Yes	Yes	Yes	Yes
Kirschen et al., 2005 [73]	Yes	Yes	Yes	Yes
Koch et al., 2006 [74]	Yes	Yes	Yes	Yes
Kondo et al., 2005 [75]	Yes	Yes	Yes	Yes
Kuhn et al., 2013 [76]	Yes	Yes	Yes	Yes

Study omitted	R SPL/precuneus (18,−56,50)	L ACC/paracingulate (−2,34,24)	R insula (46,22,−2)	L PCC (−4,−38,30)
Landau et al., 2004 [77]	Yes	Yes	Yes	Yes
Miró-Padilla et al., 2019 [78]	Yes	Yes	Yes	Yes
Miró-Padilla et al., 2020 [79]	Yes	Yes	Yes	Yes
Olesen et al., 2004 [80]	Yes	Yes	Yes	Yes
Opitz et al., 2014 [81]	Yes	Yes	Yes	Yes
Salminen et al., 2016 [118]	Yes	Yes	Yes	Yes
Schmicker et al., 2016 [82]	Yes	Yes	Yes	Yes
Schneiders et al., 2012 [83]	Yes	Yes	Yes	Yes
Schweizer et al., 2013 [84]	Yes	Yes	Yes	Yes
Takeuchi et al., 2011 [85]	Yes	Yes	Yes	Yes
Vartanian et al., 2013 [119]	Yes	Yes	Yes	Yes
Wagner et al., 2021 [86]	Yes	Yes	Yes	Yes
Westerberg et al., 2007 [87]	Yes	Yes	Yes	Yes
Zimmer et al., 2012 [88]	Yes	Yes	Yes	Yes
Dahlin et al., 2008 [89]	Yes	Yes	Yes	Yes
Bless et al., 2014 [90]	Yes	Yes	Yes	Yes
Lorenz et al., 2015 [91]	Yes	Yes	Yes	Yes
Lee et al., 2012 [92]	Yes	Yes	Yes	Yes
Sacco et al., 2022 [93]	Yes	Yes	Yes	Yes
Zhao et al., 2021 [94]	Yes	Yes	Yes	Yes

Study omitted	R SPL/precuneus (18,−56,50)	L ACC/paracingulate (−2,34,24)	R insula (46,22,−2)	L PCC (−4,−38,30)
Hummer et al., 2019 [95]	Yes	Yes	Yes	Yes
Beauchamp et al., 2016 [96]	Yes	Yes	Yes	Yes
Flegal et al., 2019 [97]	Yes	Yes	Yes	Yes
Chen et al., 2020 [98]	Yes	Yes	Yes	Yes
Wu et al., 2021 [113]	Yes	Yes	Yes	Yes
Nęcka et al., 2021 [112]	Yes	Yes	Yes	Yes

Note. Rows are unique study-level jackknife units, and columns are significant clusters from the young-adult main SDM analysis. Yes indicates that the cluster remained significant after the omitted study was removed. Multiple dependent task contrasts from the same study were removed together. Threshold: voxel-wise $p < 0.005$, absolute SDM-Z > 1, and cluster extent $k \geq 100$ voxels in MNI space. Bracketed citation numbers refer to the main-text reference list.

Supplementary Table S9. Behavioral sensitivity analysis using alternative pre–post correlation assumptions.

Assumed r	Analysis	k	Hedges' g	SE	95% CI	z	p
0	Overall	51	0.546	0.067	[0.414, 0.678]	8.103	<0.001
0	Children/adolescents	12	0.623	0.135	[0.359, 0.887]	4.624	<0.001
0	Young adults	40	0.516	0.076	[0.367, 0.665]	6.797	<0.001
0.10	Overall	51	0.546	0.067	[0.414, 0.678]	8.115	<0.001
0.10	Children/adolescents	12	0.621	0.134	[0.358, 0.885]	4.624	<0.001
0.10	Young adults	40	0.518	0.076	[0.369, 0.667]	6.809	<0.001
0.30	Overall	51	0.546	0.067	[0.415, 0.678]	8.143	<0.001
0.30	Children/adolescents	12	0.618	0.134	[0.356, 0.881]	4.618	<0.001
0.30	Young adults	40	0.519	0.076	[0.370, 0.668]	6.839	<0.001
0.50	Overall	51	0.545	0.067	[0.414, 0.675]	8.176	<0.001
0.50	Children/adolescents	12	0.614	0.133	[0.353, 0.876]	4.603	<0.001
0.50	Young adults	40	0.518	0.075	[0.370, 0.666]	6.877	<0.001
0.70	Overall	51	0.541	0.066	[0.412, 0.670]	8.208	<0.001
0.70	Children/adolescents	12	0.609	0.133	[0.349, 0.870]	4.579	<0.001
0.70	Young adults	40	0.515	0.074	[0.369, 0.661]	6.921	<0.001

Note. Assumed r is the pre–post correlation parameter used to re-estimate effect sizes and sampling variances for formula-derived single-group pre–post effects. The sensitivity analysis was rerun on the same 51-study, 205-effect-size dataset as the primary model. k indicates unique studies. Effect directions were unchanged across all assumptions.

Supplementary Table S10. Behavioral funnel-plot, small-study-effect, and correction checks.

	k	Unadjusted Hedges' g [95% CI]	Egger p	Trim-and-fill Hedges' g [95% CI]	PEESE Hedges' g [95% CI]
Overall	51	0.532 [0.402, 0.662]	0.751	0.532 [0.402, 0.662]; k0 = 0	0.473 [0.295, 0.652]
Children/adolescents	12	0.617 [0.336, 0.898]	0.401	0.617 [0.336, 0.898]; k0 = 0	0.690 [0.272, 1.108]
Young adults	40	0.504 [0.362, 0.646]	0.228	0.504 [0.362, 0.646]; k0 = 0	0.375 [0.179, 0.570]

Note. Funnel plots, Egger tests, trim-and-fill, and PEESE estimates were conducted after aggregating dependent behavioral effects within each study using inverse-variance weights; results are descriptive because the primary model retained dependent effect sizes in a three-level structure.

Supplementary Table S11. SDM cluster-level funnel-plot and Egger diagnostics for main-analysis clusters.

Population	Main-analysis cluster	Bias	t(df)	p
Overall	R SPL (18,−64,54)	0.05	0.35 (135)	0.727
Overall	R OP/V1 (14,−94,6)	0.23	1.15 (135)	0.251
Overall	L cerebellum lobule IV/V (−6,−50,0)	0.31	2.44 (135)	0.016
Overall	R posterior STG (60,−32,10)	0.09	0.62 (135)	0.539
Overall	L ACC/paracingulate (−2,28,28)	0.51	3.49 (135)	0.001
Overall	R insula (44,20,−2)	0.50	2.67 (135)	0.009
Overall	L PCC (−2,−34,30)	0.85	4.34 (135)	<0.001
Children/adolescents	R cuneus (16,−96,6)	−0.54	−1.13 (29)	0.269
Children/adolescents	R MFG (32,50,22)	0.55	0.98 (29)	0.337
Children/adolescents	L OP/V1 (−14,−92,0)	−0.44	−0.94 (29)	0.355
Children/adolescents	R IPL (44,−34,46)	0.01	0.03 (29)	0.977
Children/adolescents	L SMA (0,18,48)	0.40	1.08 (29)	0.290
Children/adolescents	L MFG (−34,28,30)	0.41	1.26 (29)	0.218
Children/adolescents	L insula (−42,16,−4)	0.54	1.47 (29)	0.152
Young-adult	R SPL/precuneus (18,−56,50)	−0.03	−0.20 (104)	0.844
Young-adult	L ACC/paracingulate (−2,34,24)	0.75	3.76 (104)	<0.001
Young-adult	R insula (46,22,−2)	0.59	2.46 (104)	0.016
Young-adult	L PCC (−4,−38,30)	0.98	3.98 (104)	<0.001

Note. Funnel plots and Egger tests were generated using SDM after adding an AllStudies filter column to temporary copies of the SDM tables; original SDM input tables were not modified. These diagnostics summarize cluster-level asymmetry of extracted study-map estimates and should not be interpreted as conventional trim-and-fill correction of scalar effect sizes.

Supplementary Table S12. Sample-level sensitivity analysis of dependent SDM contrasts.

Direction	Region	Primary analysis				Sample-level analysis			
		MNI coordinate	SDM-Z	p	Voxels	MNI coordinate	SDM-Z	p	Voxels
Positive	R occipital/cuneus	14,−94,6	1.222	<0.001	769	16,−96,12	1.438	<0.001	1293
Positive	R posterior STG	60,−32,10	1.061	<0.001	562	58,−32,8	1.118	<0.001	794
Positive	L cerebellar lobule IV/V	−6,−50,0	1.131	<0.001	639	−4,−48,−2	1.010	0.001	441
Positive	R SPL	18,−64,54	1.522	<0.001	771	18,−64,52	1.256	<0.001	337
Negative	ACC/paracingulate	−2,28,28	−2.930	<0.001	3054	−2,34,24	−2.892	<0.001	1768
Negative	R insula	44,20,−2	−2.484	<0.001	989	44,20,−2	−2.676	<0.001	651
Negative	PCC	−2,−34,30	−1.888	0.002	330	−2,−36,32	−2.138	0.001	292

Note. The sample-level analysis comprised 77 maps derived from the 137 eligible contrasts. Dependent contrasts were combined using SDM Combine Images with an assumed correlation of r = 0.50, followed by re-preprocessing with 50 Monte Carlo randomizations. Both analyses used voxel-wise $p < 0.005$, $|SDM\text{-}Z| > 1$, and cluster extent $k \geq 100$ voxels in MNI space. The Dice coefficient between the thresholded primary and sample-level maps was 0.748. ACC = anterior cingulate cortex; L/R = left/right; PCC = posterior cingulate cortex; SPL = superior parietal lobule; STG = superior temporal gyrus.

Supplementary Figures

Supplementary Figure S1. Contour-enhanced funnel plots for the behavioral meta-analysis.

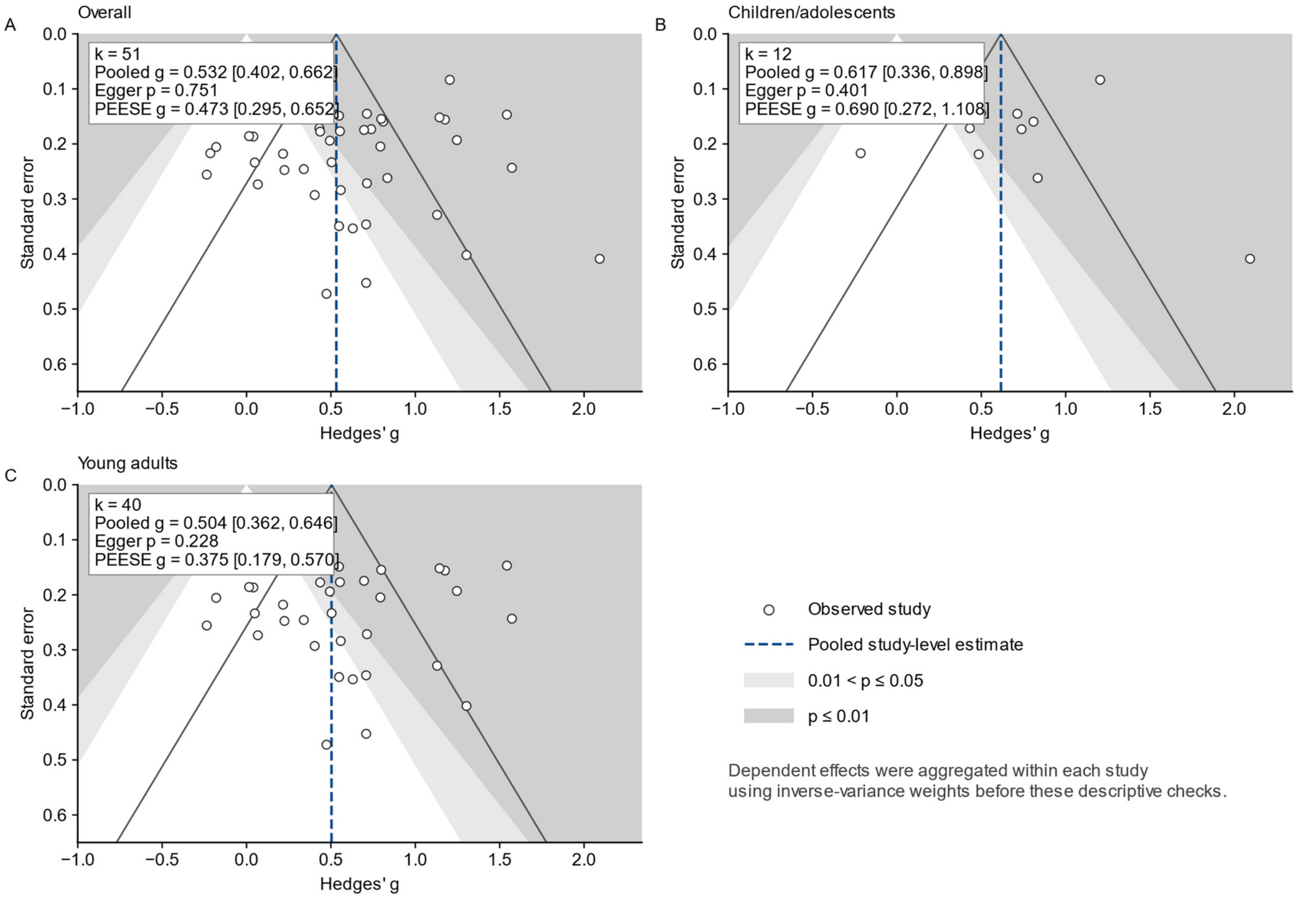

Note. Funnel plots are based on study-level effects after inverse-variance aggregation within each study. White circles indicate observed studies. No studies were imputed by the trim-and-fill procedure in any behavioral analysis (k0 = 0).

Supplementary Figure S2. Cluster-level SDM funnel plots for significant main-analysis clusters.

Supplementary Figure S2a. Overall sample.

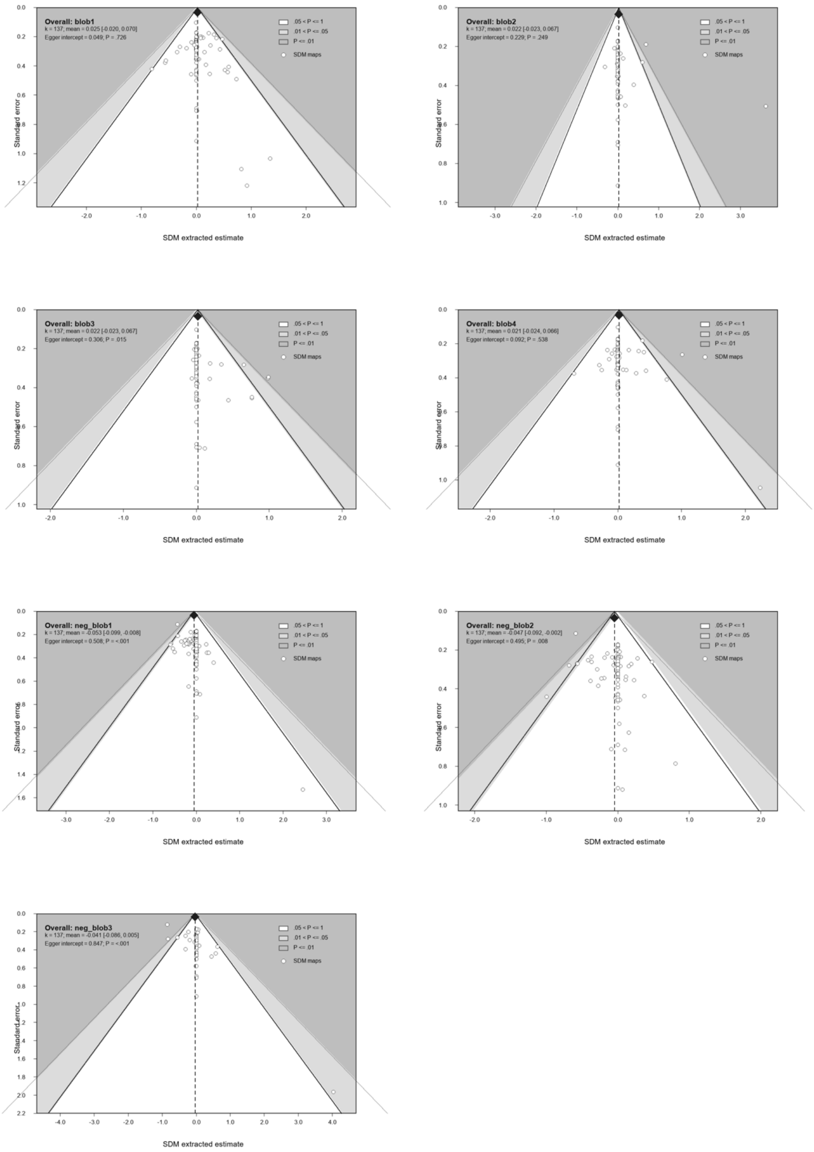

Supplementary Figure S2b. Children/adolescents sample.

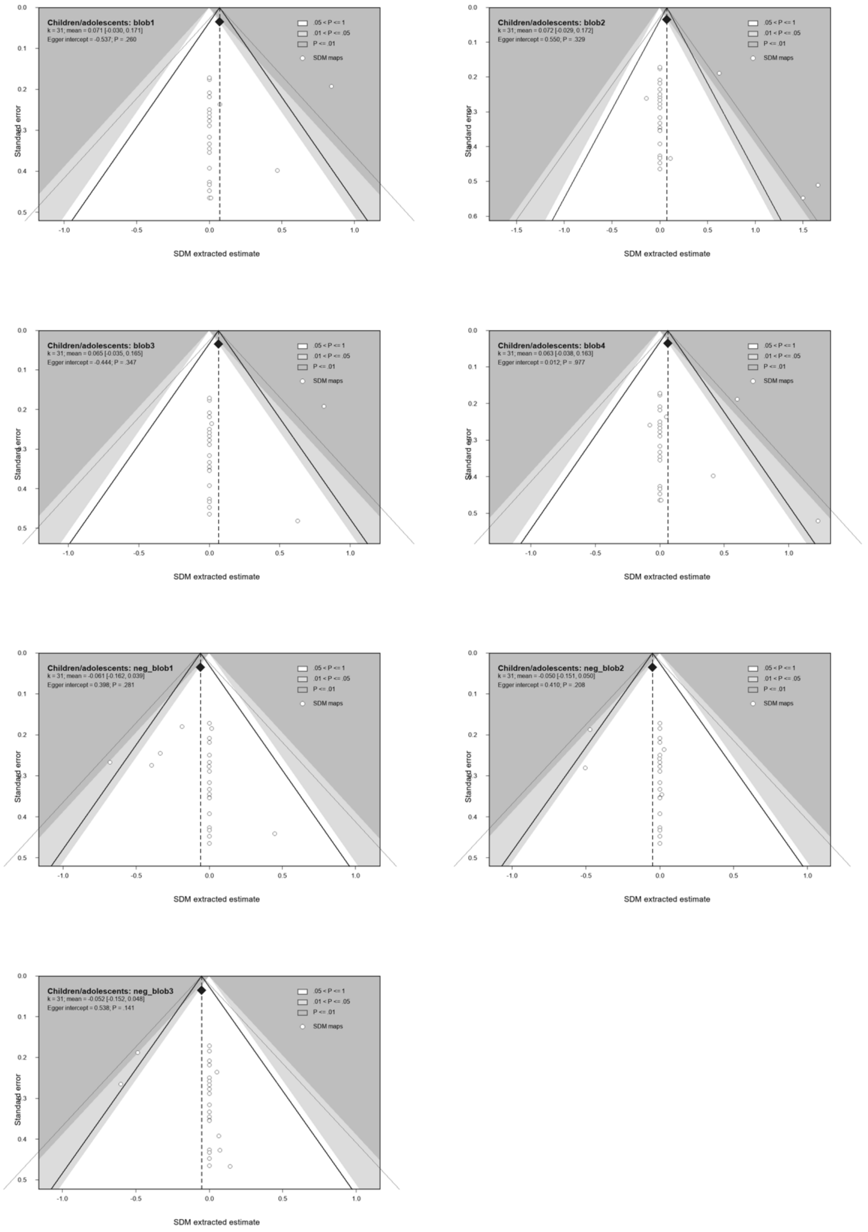

Supplementary Figure S2c. Young-adult sample.

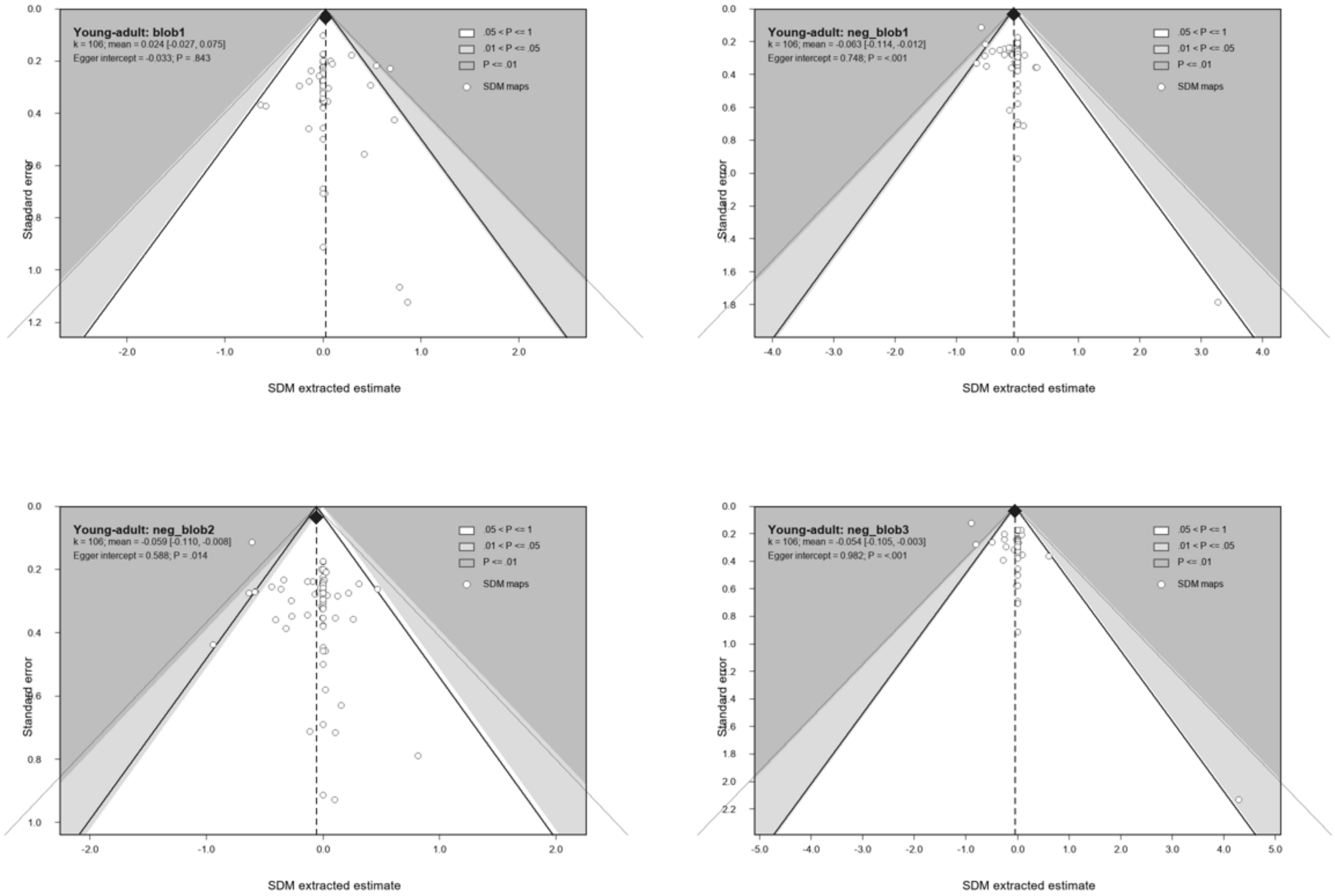

Note. Supplementary Figures S2a–S2c show the overall, children/adolescents, and young-adult main SDM analyses, respectively. The corresponding Egger statistics are summarized in Supplementary Table S11.
